# Gas Biopsy of Pleural Effusion to Diagnose Lung Cancer by Mass Spectrometry

**DOI:** 10.1002/mco2.70787

**Published:** 2026-06-04

**Authors:** Wenting Liu, Jian Qi, Yajing Chu, Jijuan Zhou, Yue Liu, Li Ke, Xiangxue Zheng, Yan Lu, Dianlong Ge, Yannan Chu, Hongzhi Wang

**Affiliations:** ^1^ Hefei Cancer Hospital of CAS Institute of Health and Medical Technology, Hefei Institutes of Physical Science Chinese Academy of Sciences (CAS) Hefei Anhui China; ^2^ Science Island Branch Graduate School of the University of Science and Technology of China Hefei Anhui China; ^3^ Department of Geriatric Medicine The First Affiliated Hospital of the University of Science and Technology of China Division of Life Sciences and Medicine University of Science and Technology of China Hefei Anhui China; ^4^ Anhui Province Key Laboratory of Medical Physics and Technology Hefei Anhui China; ^5^ Key Laboratory of Biodiversity Conservation and Characteristic Resource Utilization in Southwest Anhui School of Life Sciences Anqing Forestry Technology Innovation Research Institute Anqing Normal University Anhui China; ^6^ Department of Thoracic Surgery The First Affiliated Hospital of the University of Science and Technology of China Division of Life Sciences and Medicine University of Science and Technology of China Hefei Anhui China

**Keywords:** gas biopsy, lung cancer, pleural effusion, tumor tissue, volatile organic compounds

## Abstract

Lung cancer (LC) is a leading cause of cancer mortality worldwide, frequently complicated by malignant pleural effusion (MPE). This study aimed to investigate volatile organic compounds (VOCs) in pleural effusion to identify metabolic biomarkers for LC. A total of 107 LC‐related MPE and 112 benign pleural effusion (BPE) patients were enrolled and divided into discovery (MPE: *n* = 75; BPE: *n* = 78) and validation (MPE: *n* = 32; BPE: *n* = 34) cohorts. Paired tumor and adjacent nontumor tissues from 14 LC patients formed an independent cohort. Differential VOCs were identified using criteria of *q* < 0.05, |log_2_ fold change| > 1, and VIP > 1.5 in both the pleural effusion discovery cohort and the tissue cohort. Hexanal was identified as the only LC‐associated VOC present across both compartments. In the validation cohort, hexanal demonstrated strong diagnostic performance in distinguishing MPE from BPE (AUC = 0.8339; sensitivity 75.00%; specificity 65.71%). Single‐cell analysis indicated that hexanal accumulation is linked to fatty acid metabolism reprogramming. This study supports pleural effusion‐based gas biopsy as a promising LC diagnostic approach.

## Graphical abstract

HS‐SPME–GC–MS profiling of pleural effusion revealed distinct volatile metabolic features between lung cancer‐associated malignant pleural effusion and benign pleural effusion. Cross‐matrix integration with paired lung cancer tissues identified hexanal as the only shared differential VOC. Independent validation supported the diagnostic potential of hexanal, highlighting pleural effusion‐based gas biopsy as a promising approach for lung cancer diagnosis.

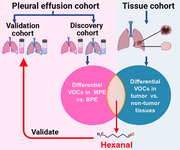

## 1 Introduction

Lung cancer (LC) remains one of the most common and deadly malignancies worldwide. Because early‐stage LC often lacks specific clinical manifestations, many patients are diagnosed only after distant metastasis has occurred, resulting in a 5‐year survival rate of less than 20% [1, 2]. This unfavorable clinical reality underscores the urgent need for novel and effective approaches to LC detection [3].

Gas biopsy, a noninvasive strategy that interrogates volatile organic compounds (VOCs) to characterize disease‐associated metabolic alterations, has emerged as a promising approach in LC research [4]. Increasing evidence suggests that cancer‐related VOCs are closely linked to tumor metabolic reprogramming [4, 5, 6]. In particular, oxidative stress within the tumor microenvironment can drive lipid peroxidation (LPO) of polyunsaturated fatty acids (PUFAs), leading to the generation of volatile aldehydes and other low‐molecular‐weight compounds [7]. By capturing these metabolically informative volatile molecules, gas biopsy provides new opportunities for biomarker discovery and molecular characterization in LC [8].

However, LC‐related VOC biomarkers reported to date remain highly heterogeneous, and no robust diagnostic consensus has yet been established [9, 10]. This heterogeneity likely reflects the combined influence of biological and technical factors, including sample type, environmental exposure, dietary habits, interindividual metabolic variation, analytical platforms, sample pretreatment procedures, and data‐processing strategies [8, 10]. Together, these factors may obscure tumor‐derived metabolic signals, thereby limiting the reproducibility of VOC‐based biomarker studies and hindering their clinical translation. These challenges highlight the importance of selecting an appropriate sample matrix and clarifying the biological origin of candidate VOC biomarkers in LC‐related volatilomic research [8].

Malignant pleural effusion (MPE) is a common clinical complication of LC and has unique methodological value in LC research [11]. As a proximal fluid derived from the pleural cavity, MPE represents a local anatomical and biological compartment in which tumor cells continuously interact with the surrounding microenvironment [11, 12]. Compared with other body fluids, MPE may better capture the biological characteristics of pleural metastatic lesions and is more likely to be enriched in locally derived tumor cells, nucleic acids, proteins, and metabolites [13, 14]. Therefore, MPE is not only an important manifestation of LC progression but also an ideal sample for investigating pleural metastasis, identifying local diagnostic biomarkers, and exploring novel molecular diagnostic strategies [14].

**FIGURE 1:**
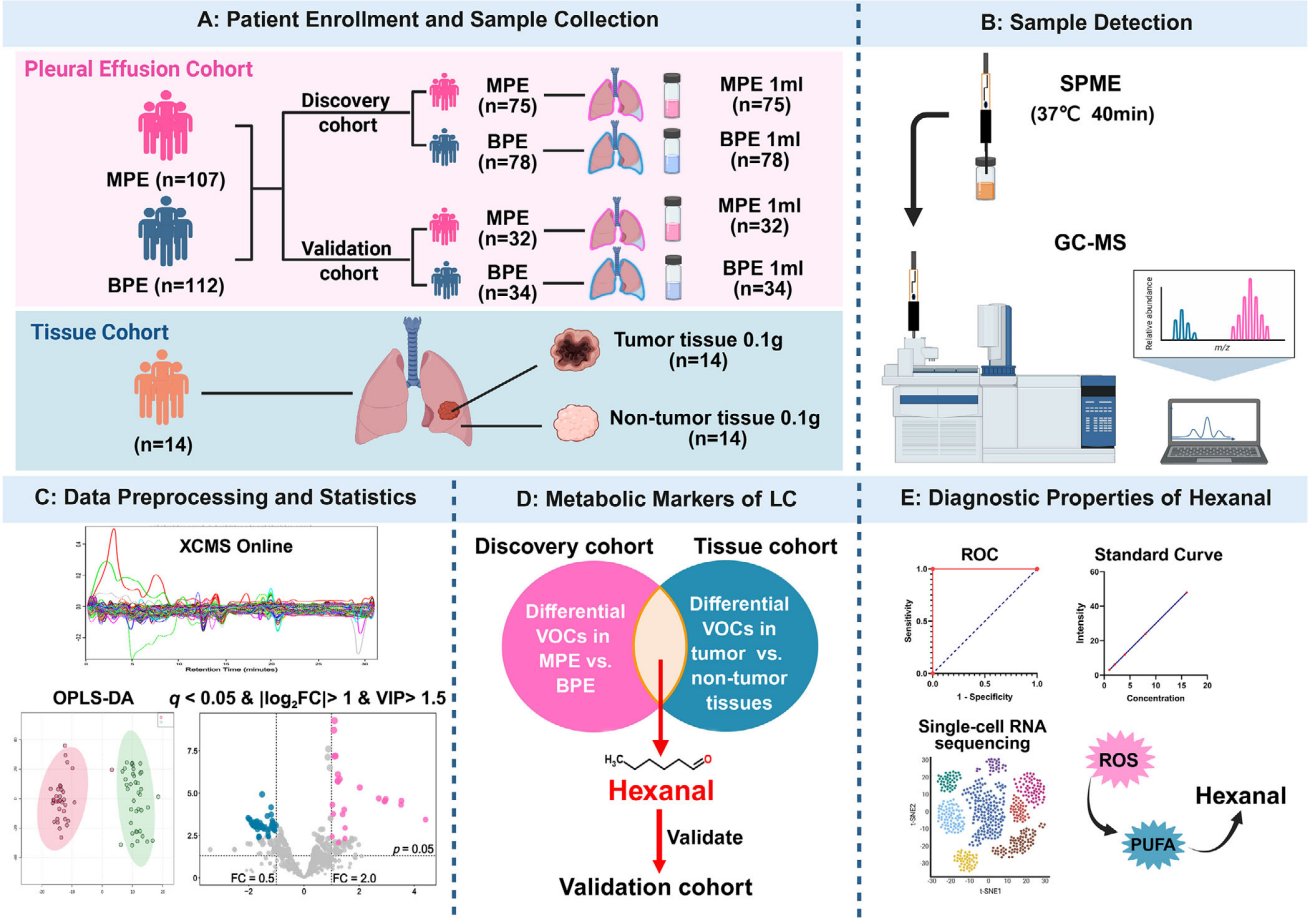
Study design and workflow for the identification and validation of VOC biomarkers in pleural effusion and LC. (A) Patient enrollment and sample collection. The pleural effusion cohort included 219 patients, comprising 107 patients with MPE and 112 patients with BPE. These patients were randomly divided into a discovery cohort (MPE, *n* = 75; BPE, *n* = 78) and a validation cohort (MPE, *n* = 32; BPE, *n* = 34). For VOC analysis, 1 mL of pleural effusion was collected from each participant. In parallel, the tissue cohort included 14 patients with LC, from whom paired tumor tissues and adjacent nontumor tissues (0.1 g each) were collected. (B) Sample detection. VOCs in pleural effusion and tissue samples were extracted using HS‐SPME at 37°C for 40 min, followed by GC–MS. (C) Data preprocessing and statistical analysis. Raw GC–MS data were processed using XCMS Online. Group discrimination and differential feature screening were performed using OPLS‐DA and the following criteria: *q* < 0.05, |log_2_FC| > 1, and VIP > 1.5. (D) Identification of metabolic markers of LC. Differential VOCs identified in the pleural effusion discovery cohort (MPE vs. BPE) were intersected with those identified in the tissue cohort (tumor vs. nontumor tissues). Hexanal was identified as the only overlapping LC‐associated VOC and was further evaluated in the validation cohort. (E) Diagnostic and biological evaluation of hexanal. The diagnostic performance of hexanal was assessed by ROC analysis and quantitative standard curve validation. Single‐cell RNA sequencing analysis further suggested that hexanal accumulation may be associated with ROS‐related lipid peroxidation of PUFAs in LC tissues. BPE, benign pleural effusion; FC, fold change; GC–MS, gas chromatography–mass spectrometry; HS‐SPME, headspace solid‐phase microextraction; LC, lung cancer; MPE, malignant pleural effusion; OPLS‐DA, orthogonal partial least squares‐discriminant analysis; PUFAs, polyunsaturated fatty acids; ROC, receiver operating characteristic; ROS, reactive oxygen species; VIP, variable importance in projection; VOC, volatile organic compound. Created with BioRender.com.

Gas chromatography–mass spectrometry (GC–MS), with its high sensitivity and resolution, has become a key analytical platform for VOC research [15]. Previous GC–MS‐based studies have identified differential metabolites between MPE and benign pleural effusion (BPE), suggesting that pleural fluid metabolomics may help distinguish MPE from BPE [16, 17]. Nevertheless, reproducibility across studies remains limited, and many reports have been constrained by relatively small sample sizes [16, 17]. More importantly, previous investigations have largely focused on the metabolic characteristics of pleural effusion, whereas the relationship between MPE‐associated VOCs and the metabolic features of LC has not been systematically explored. To address this gap, we applied headspace solid‐phase microextraction (HS‐SPME) coupled with GC–MS to compare VOC profiles between LC‐associated MPE and BPE in a relatively large cohort, which was randomly stratified into discovery and validation cohorts at a ratio of 7:3. To clarify the relevance of candidate MPE‐specific VOCs to LC and to improve their biological interpretability, we further profiled volatile metabolites in paired LC tissues and adjacent nontumor tissues. By integrating tissue and pleural effusion data through intersection analysis and directional consistency analysis of differential metabolites, we aimed to identify VOC biomarkers that arise from local tumor‐associated metabolic abnormalities and are consistently enriched in pleural effusion, thereby facilitating biological source tracing and cross‐validation of fluid‐based biomarkers. Through this integrative pleural effusion–tissue framework, we sought to identify LC‐related VOC biomarkers with both diagnostic value and biological plausibility (Figure 1).

## 2 Results

### 2.1 Differential VOCs in the Pleural Effusion Discovery Cohort: MPE Versus BPE

The purpose of this analysis was to determine whether the VOC profile of pleural effusion could distinguish LC‐associated MPE from BPE and to identify candidate VOCs associated with MPE. To this end, HS‐SPME–GC–MS data from 153 pleural effusion samples in the discovery cohort were subjected to untargeted preprocessing using XCMS Online, followed by multivariate modeling, statistical screening, and VOC annotation.

**FIGURE 2:**
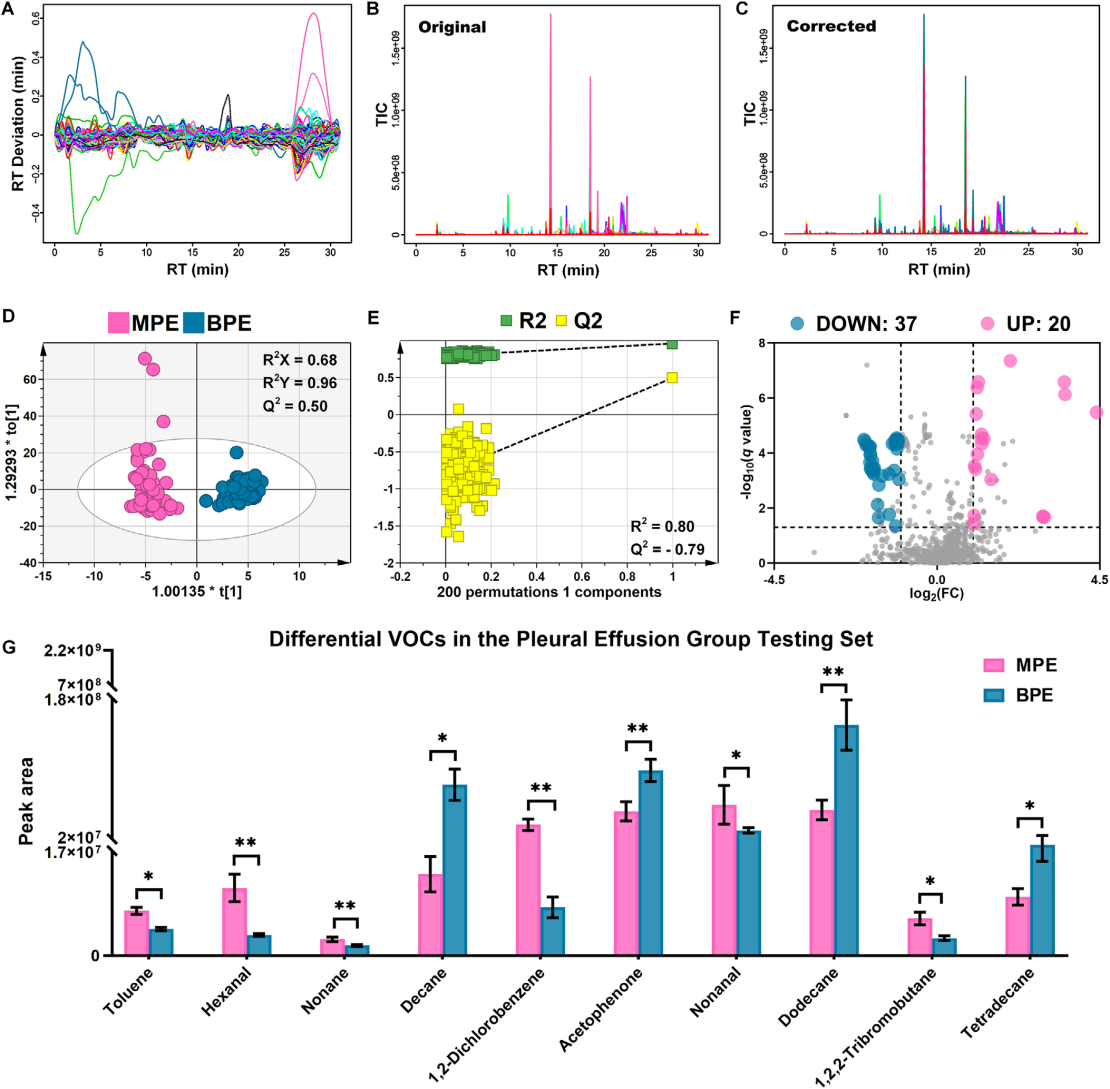
Identification of differential VOCs in the pleural effusion discovery cohort. (A) RT alignment curve after XCMS Online preprocessing. (B and C) Representative TICs before and after preprocessing. (D) OPLS‐DA score plot showing separation between the MPE and BPE groups. (E) Permutation test of the OPLS‐DA model. (F) Volcano plot of differential characteristic ions screened using *q* < 0.05, |log_2_FC| > 1, and VIP > 1.5. (G) Relative peak areas of the 10 differential VOCs identified in the pleural effusion discovery cohort. Data are presented as mean ± SEM. *Q < 0.05, **Q < 0.01. BPE, benign pleural effusion; FC, fold change; MPE, malignant pleural effusion; OPLS‐DA, orthogonal partial least squares‐discriminant analysis; RT, retention time; TIC, total ion chromatogram; VIP, variable importance in projection; VOC, volatile organic compound.

After preprocessing, 888 characteristic peaks were detected in the pleural effusion discovery cohort. The retention time alignment curve showed that the retention time deviation was maintained within ±0.40 min, supporting acceptable reproducibility and stability during GC–MS acquisition and data preprocessing (Figure 2A). Representative total ion chromatograms (TICs) before and after preprocessing further demonstrated improved baseline smoothness, reduced background noise, and enhanced consistency of ion signals after data correction and alignment (Figure 2B,C). To evaluate whether the global VOC profile differed between MPE and BPE, orthogonal partial least squares‐discriminant analysis (OPLS‐DA) was performed. The score plot showed clear separation between the two groups, with model parameters of *R* ^2^ *X* = 0.68, *R* ^2^ *Y* = 0.96, and *Q* ^2^ = 0.50 (Figure 2D). The 200‐times permutation test yielded a negative *Q* ^2^ intercept, indicating that the model was not overfitted and that the observed separation was unlikely to be caused by random class assignment (Figure 2E).

Differential characteristic ions were then screened using combined criteria of false discovery rate (FDR)‐adjusted *q* < 0.05, |log_2_ fold change (FC)| > 1, and variable importance in projection (VIP) > 1.5, as visualized in the volcano plot (Figure 2F). A total of 57 differential characteristic ions met these criteria and are listed in Table S1. These ions were further annotated using the National Institute of Standards and Technology (NIST) 14.0 database with a reverse search index (RSI) > 800, together with retention index (RI) comparison against reported reference values. Subsequent nonparametric comparison of annotated VOCs between MPE and BPE identified 10 significantly differential VOCs: toluene, hexanal, nonane, decane, 1,2‐dichlorobenzene, acetophenone, nonanal, dodecane, 1,2,2‐tribromobutane, and tetradecane (*Q* < 0.05; Figure 2G). Figure 2G shows the relative peak areas of these VOCs between the two groups, illustrating their differential abundance patterns in MPE and BPE. Detailed information, including retention time, Chemical Abstracts Service number, RSI, RI, characteristic ions, *q* values, log_2_FC values, and VIP values, is summarized in Table 1.

**TABLE 1:** Differential VOCs in the pleural effusion discovery cohort.

	VOCs	RT	*Q* value^a^	CAS	RSI	RI^a^	RI^b^	Characteristic	*q* Value^b^	log_2_ (FC)	VIP
		(min)						ions			
1	Toluene ↑	8.73	0.01	108‐88‐3	931	−	770	M65T9	4.15E−07	1.11	3.08
								M63T9_2	3.76E−06	1.09	2.96
								M90T9	1.11E−04	1.12	2.74
2	Hexanal ↑	10.35	1.73E−05	66‐25‐1	824	838	826	M72T10	0.04	1.01	1.66
3	Nonane ↑	12.56	1.05E−03	111‐84‐2	924	902	900	M59T13	2.63E−07	1.14	2.73
4	Decane ↓	15.98	0.01	124‐18‐5	934	1001	1000	M143T16	7.46E−03	−1.64	1.52
								M142T16	0.02	−1.62	1.53
5	1,2‐Dichlorobenzene ↑	17.60	4.04E−07	95‐50‐1	909	1086	1076	M146T18	0.02	2.93	2.14
								M148T18	0.02	2.92	2.14
								M150T18	0.02	2.97	2.17
6	Acetophenone ↓	20.21	2.08E−03	98‐86‐2	918	1152	1094	M120T20	2.85E−05	−1.12	1.77
								M51T20	3.26E−05	−1.11	1.85
								M77T20	3.26E−05	−1.10	1.81
								M105T20	3.38E−05	−1.07	1.74
								M121T20	3.43E−05	−1.16	1.84
								M78T20	3.72E−05	−1.09	1.82
								M50T20	3.79E−05	−1.13	1.89
								M74T20	4.56E−05	−1.24	1.76
								M106T20	4.56E−05	−1.08	1.77
								M52T20	4.90E−05	−1.16	1.79
								M76T20	6.93E−05	−1.11	1.83
7	Nonanal ↑	20.74	0.04	124‐19‐6	914	1163	1142	M82T21	0.02	1.03	1.52
8	Dodecane ↓	22.43	2.92E−03	112‐40‐3	929	1201	1200	M171T22_2	3.72E−05	−1.99	1.86
								M86T22	5.87E−05	−1.84	1.77
								M72T22	6.11E−05	−1.87	1.77
								M71T22	1.12E−04	−1.84	1.76
								M85T22	1.13E−04	−1.85	1.77
								M113T22	1.93E−04	−1.78	1.72
								M141T22_1	2.18E−04	−1.86	1.78
								M84T22	3.49E−04	−1.73	1.74
								M99T22_1	3.51E−04	−1.84	1.75
								M112T22	3.84E−04	−1.75	1.74
								M126T22	3.88E−04	−1.77	1.77
								M56T22	4.03E−04	−1.83	1.76
								M127T22	5.09E−04	−1.77	1.73
								M70T22	5.81E−04	−1.75	1.73
								M140T22_2	1.45E−03	−1.61	1.70
								M172T22_2	3.26E−05	−2.02	1.89
								M170T22	5.18E−05	−1.96	1.86
								M57T22	5.33E−05	−1.91	1.80
9	1,2,2‐ Tribromobutane ↑	24.55	0.01	3675‐69‐2	866	1273	1270	M175T25	4.43E−08	2.03	2.34
10	Tetradecane ↓	28.13	0.01	629‐59‐4	901	1401	1400	M199T28	0.02	−1.44	1.67

↑ indicates that the peak area of the VOC in MPE was significantly higher than that in BPE; ↓ indicates that the peak area of the VOC in MPE was significantly lower than that in BPE. *Q* Value^a^ refers to the FDR‐adjusted *Q* value of the peak area for the corresponding differential VOC derived from the Mann–Whitney *U* test between MPE and BPE. *q* Value^b^ refers to the FDR‐adjusted *q* value of the peak area for the corresponding characteristic ion derived from the Mann–Whitney *U* test between MPE and BPE. RI^a^, experimentally calculated retention index; RI^b^, reported reference retention index. “–” indicates not available. Abbreviations: BPE, benign pleural effusion; CAS, Chemical Abstracts Service; FC, fold change; MPE, malignant pleural effusion; RSI, reverse search index; RT, retention time; VIP, variable importance in projection; VOC, volatile organic compound.

Together, these results demonstrate that pleural effusion from LC‐associated MPE patients has a distinct VOC profile compared with BPE and provide a panel of differential VOCs for subsequent cross‐matrix biomarker screening.

### 2.2 Differential VOCs in the Tissue Cohort: Tumor Tissue Versus Nontumor Tissue

The purpose of the tissue cohort analysis was to determine whether LC tumor tissues contained VOC alterations consistent with tumor‐associated metabolic reprogramming and to provide a biological reference for identifying pleural effusion VOCs with potential tumor relevance. Therefore, paired tumor and adjacent nontumor lung tissue samples from 14 LC patients were analyzed by HS‐SPME‐GC–MS, followed by the same untargeted preprocessing and differential screening strategy used for the pleural effusion cohort.

**FIGURE 3:**
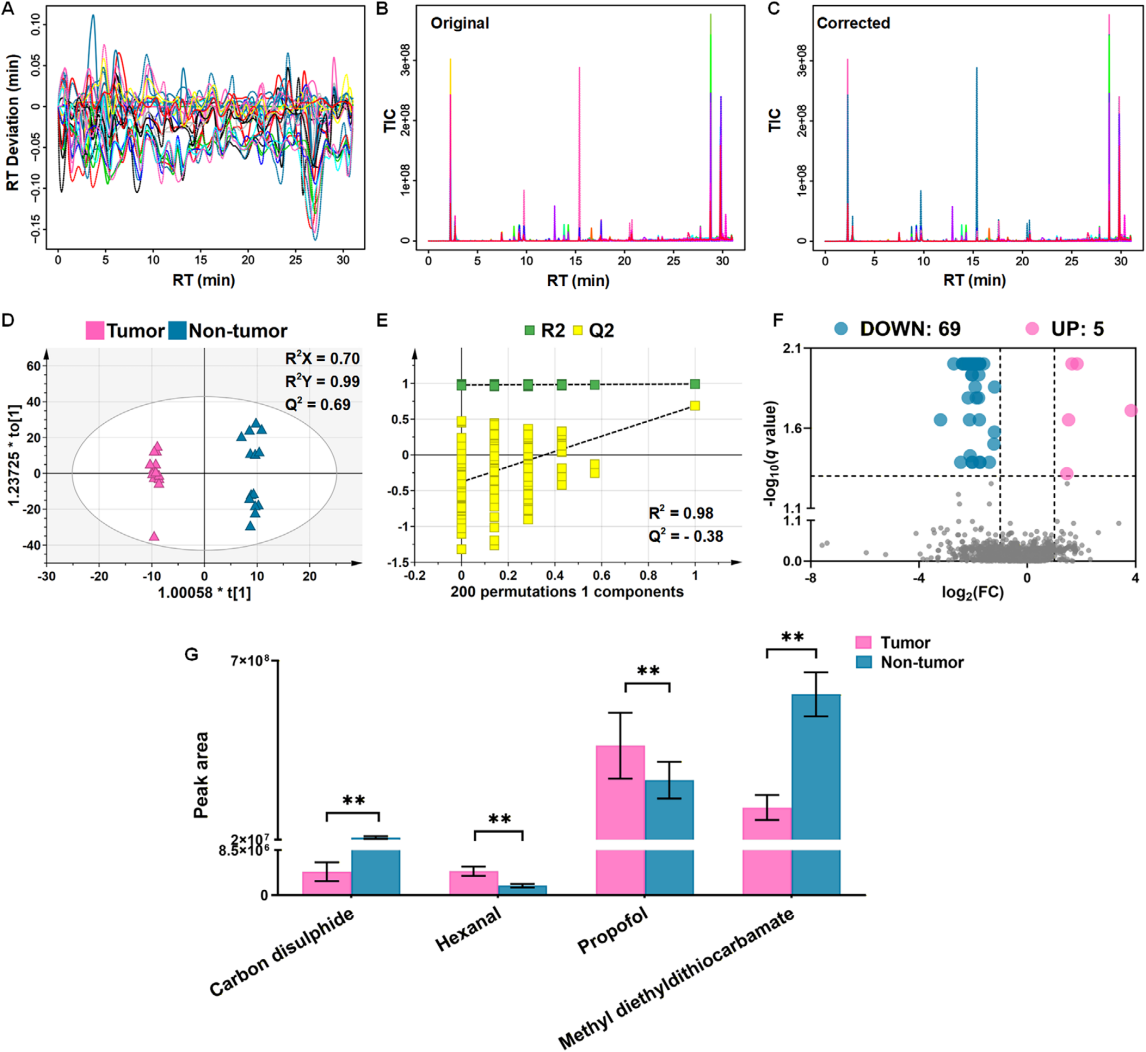
Differential VOC profiles in the tissue cohort. (A) RT alignment curve after XCMS Online preprocessing. (B and C) Representative TICs before and after preprocessing. (D) OPLS‐DA score plot showing separation between tumor and adjacent nontumor tissues. (E) Permutation test of the OPLS‐DA model. (F) Volcano plot of differential characteristic ions screened using *q* < 0.05, |log_2_FC| > 1, and VIP > 1.5. (G) Relative peak areas of the four differential VOCs identified in the tissue cohort. Data are presented as mean ± SEM. **Q < 0.01. FC, fold change; GC–MS, gas chromatography–mass spectrometry; OPLS‐DA, orthogonal partial least squares‐discriminant analysis; *q*, false discovery rate‐adjusted *p* value; RT, retention time; SEM, standard error of the mean; TIC, total ion chromatogram; VIP, variable importance in projection; VOC, volatile organic compound.

XCMS Online preprocessing of GC–MS data from 28 tissue samples identified 1204 characteristic peaks. The retention time alignment curve showed that the deviation was stable within ±0.15 min, indicating good analytical reproducibility for tissue VOC profiling (Figure 3A). Representative TICs before and after preprocessing showed correction of baseline drift, reduction of background noise, and improved consistency of signal response after data processing (Figure 3B,C). OPLS‐DA was then used to evaluate the global separation between tumor and adjacent nontumor tissues. The score plot demonstrated clear discrimination between the two tissue types, with *R* ^2^ *X* = 0.70, *R* ^2^ *Y* = 0.99, and *Q* ^2^ = 0.69 (Figure 3D). A 200‐times permutation test showed a *Q* ^2^ intercept < 0, supporting the robustness of the model and suggesting no obvious overfitting (Figure 3E).

Using the predefined criteria of *q* < 0.05, |log_2_FC| > 1, and VIP > 1.5, 74 differential characteristic ions were identified, as shown in the volcano plot (Figure 3F) and listed in Table S2. These characteristic ions were annotated by matching mass spectra against the NIST 14.0 database with RSI > 800 and by comparing experimental RI values with reported reference values. After Mann–Whitney *U* testing with FDR correction, four significantly differential VOCs were identified between tumor and adjacent nontumor tissues: carbon disulfide, hexanal, propofol, and methyl diethyldithiocarbamate (*Q* < 0.05; Table 2). Their relative peak areas are shown in Figure 3G, which illustrates the differential abundance of these four VOCs in the tissue cohort.

**TABLE 2:** Differential VOCs in the tissue cohort.

	VOCs	RT	*Q* value^a^	CAS	RSI	RI^a^	RI^b^	Characteristic	*q* Value^b^	log_2_ (FC)	VIP
		(min)						ions			
1	Carbon disulfide ↓	2.75	1.52E−05	75‐15‐0	890	−	576	M64T3	0.04	−2.11	1.82
								M78T3	0.04	−2.04	1.72
								M80T3	0.01	−2.55	1.71
								M76T3	0.04	−2.04	1.72
2	Hexanal ↑	10.35	6.24E−03	66‐25‐1	824	837	826	M137T10	0.01	1.83	2.50
								M153T10	0.01	1.65	2.36
3	Propofol ↑	28.84	5.45E−04	2078‐54‐8	915	1424	1367	M279T29	0.01	2.31	1.76
4	Methyl diethyl‐ dithiocarbamate ↓	29.82	5.45E−04	686‐07‐7	965	1459	1377	M162T30	0.01	−2.08	2.35
								M166T30	0.01	−2.06	2.42
								M119T30	0.01	−2.17	2.54
								M136T30	0.01	−2.31	2.41
								M73T30_2	0.01	−1.83	2.41
								M104T30	0.01	−2.08	2.37
								M77T30	0.01	−2.11	2.47
								M150T30	0.01	−2.25	2.47
								M165T30	0.01	−2.09	2.43
								M70T30	0.01	−1.75	2.45
								M58T30	0.01	−1.79	2.40
								M49T30	0.01	−1.97	2.37
								M148T30_1	0.01	−2.15	2.39
								M62T30	0.01	−1.89	2.38
								M90T30	0.04	−1.75	2.06
								M100T30	0.02	−1.78	2.09
								M45T30	0.01	−1.88	2.44
								M84T30	0.01	−1.93	2.50
								M79T30	0.01	−1.96	2.41
								M78T30	0.01	−1.93	2.33
								M66T30	0.01	−2.03	2.48
								M167T30	0.01	−2.05	2.47
								M75T30	0.01	−1.95	2.43
								M130T30	0.01	−2.71	2.36
								M60T30	0.01	−1.88	2.38
								M63T30	0.02	−1.88	2.29
								M46T30	0.01	−1.79	2.26
								M117T30	0.01	−2.08	2.41
								M59T30	0.01	−1.90	2.42
								M61T30	0.01	−1.91	2.41
								M72T30	0.01	−1.93	2.44
								M91T30	0.01	−1.98	2.45
								M114T30	0.01	−2.03	2.41
								M54T30	0.01	−1.83	2.39
								M164T30	0.01	−2.10	2.44
								M163T30	0.01	−2.10	2.42
								M160T30	0.04	−1.76	1.97
								M92T30	0.01	−1.96	2.45
								M94T30	0.01	−2.26	2.42
								M93T30	0.01	−1.97	2.45
								M102T30	0.01	−2.11	2.38
								M52T30	0.01	−2.05	2.39
								M108T30	0.01	−2.40	2.28
								M118T30	0.01	−2.05	2.40
								M47T30	0.01	−1.77	2.34
							M134T30	0.02	−2.13	2.19
								M106T30	0.01	−2.16	2.11
								M74T30	0.01	−1.97	2.43
								M89T30	0.01	−1.99	2.42
								M56T30	0.01	−1.78	2.37
								M88T30	0.01	−2.01	2.44
								M116T30	0.01	−2.12	2.41
								M87T30	0.01	−1.94	2.39
								M48T30	0.01	−1.62	2.22
								M76T30	0.01	−1.92	2.29
								M86T30	0.01	−1.98	2.43
								M65T30	0.02	−1.80	2.41
								M64T30	0.01	−1.99	2.41

↑indicates that the peak area of the VOC in tumor tissue was significantly higher than that in nontumor tissue; ↓ indicates that the peak area of the VOC in tumor tissue was significantly lower than that in nontumor tissue. *Q* value^a^ refers to the FDR‐adjusted *Q* value of the peak area for the corresponding differential VOC derived from the Mann–Whitney *U* test between tumor tissue and adjacent nontumor tissue. *q* Value^b^ refers to the FDR‐adjusted *q* value of the peak area for the corresponding characteristic ion derived from the Mann–Whitney *U* test between tumor tissue and adjacent nontumor tissue. RI^a^, experimentally calculated retention index; RI^b^, reported reference retention index. “–” indicates not available.

Abbreviations: CAS, Chemical Abstracts Service; FC, fold change; RSI, reverse search index; RT, retention time; VIP, variable importance in projection; VOC, volatile organic compound.

These findings indicate that LC tumor tissues exhibit a distinct VOC profile compared with adjacent nontumor tissues. Importantly, the tissue‐derived differential VOCs provided an independent biological reference for identifying pleural effusion biomarkers more closely associated with tumor metabolic abnormalities.

### 2.3 Identification of Metabolic Biomarkers and Evaluation of Diagnostic Performance

The purpose of this section was to identify LC‐associated VOC biomarkers that were consistently altered in both pleural effusion and tumor tissues and to further evaluate their analytical reliability and diagnostic performance. To achieve this, differential VOCs identified in the pleural effusion discovery cohort were intersected with those identified in the tissue cohort, followed by quantitative method establishment, analytical validation, diagnostic testing in both discovery and validation cohorts, and comparison with conventional carcinoembryonic antigen (CEA)‐based biomarkers.

**FIGURE 4:**
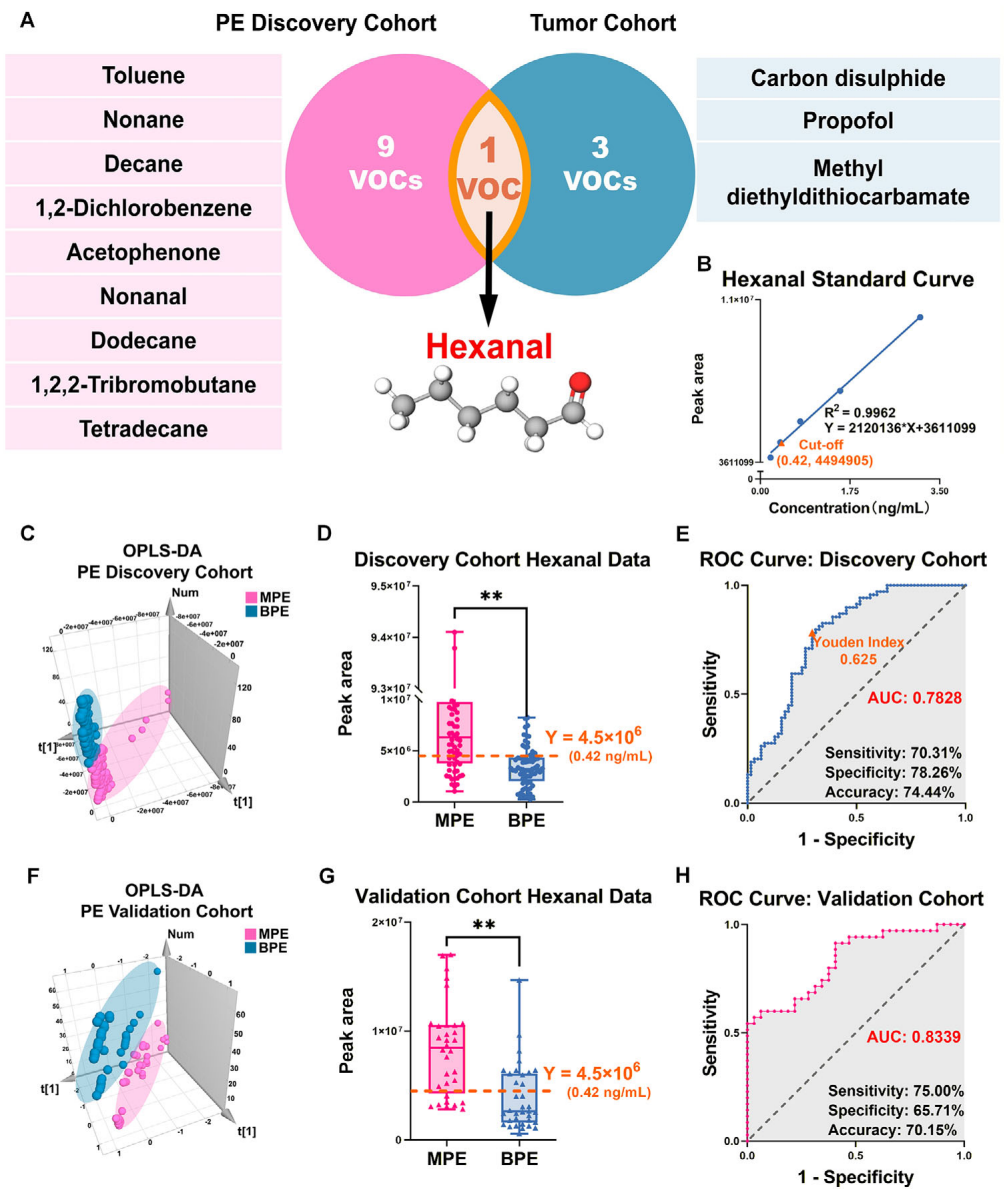
Identification of hexanal and evaluation of its diagnostic performance in the pleural effusion cohorts. (A) Overlap analysis of differential VOCs between the PE discovery cohort and the tumor cohort identified hexanal as the only shared VOC. (B) Standard curve for hexanal quantification. (C and F) OPLS‐DA score plots based on hexanal in the PE discovery and validation cohorts, respectively. (D, G) Relative peak areas of hexanal in the discovery and validation cohorts, respectively, with the diagnostic cutoff indicated by the dashed line. Data are presented as mean ± SEM. **Q < 0.01. (E and H) ROC curve analyses of hexanal in the discovery and validation cohorts, respectively. AUC, area under the curve; BPE, benign pleural effusion; MPE, malignant pleural effusion; OPLS‐DA, orthogonal partial least squares‐discriminant analysis; PE, pleural effusion; ROC, receiver operating characteristic; SEM, standard error of the mean; VOC, volatile organic compound.

Intersection analysis revealed that hexanal was the only shared differential VOC between the pleural effusion discovery cohort and the tissue cohort (Figure 4A). This result suggested that hexanal was not merely a differential pleural effusion signal but was also associated with LC tumor tissue metabolism. To support quantitative evaluation, a water‐based standard curve was established using benzaldehyde at 10 ng/mL as the internal standard and serially diluted hexanal standards ranging from 0.20 to 3.12 ng/mL. The resulting calibration equation was *Y* = 2,120,136*X* + 3,611,099, with good linearity across the tested concentration range (*R* ^2^ = 0.9962; Figure 4B).

The analytical reliability of hexanal measurement was further assessed. Storage‐time stability was evaluated using aliquoted pleural effusion samples stored at −80°C, and the results are shown in Figure S1. Intraday and interday precision were examined at low, medium, and high concentration levels. The intraday relative standard deviations (RSDs) ranged from 1.09 to 10.67% (Table S3), while the interday RSDs ranged from 4.97 to 15.02% (Table S4). The distribution of replicate measurements is further displayed in Figure S2, in which bars represent mean ± standard deviation and individual dots represent independent measurements. These results indicate acceptable reproducibility of the HS‐SPME‐GC–MS method for hexanal detection.

The diagnostic performance of hexanal was first evaluated in the pleural effusion discovery cohort. OPLS‐DA based on hexanal showed separation between MPE and BPE samples (Figure 4C). Quantitative comparison further demonstrated that hexanal levels were significantly higher in the MPE group than in the BPE group (*Q* < 0.01; Figure 4D). In Figure 4D, the dashed line indicates the diagnostic cutoff determined from receiver operating characteristic (ROC) analysis. ROC curve analysis yielded an area under the curve (AUC) of 0.7828, with a maximum Youden index of 0.625. The optimal cutoff was a peak area of 4.5 × 10^6^, corresponding to a concentration of 0.42 ng/mL, which achieved a sensitivity of 70.31%, specificity of 78.26%, and overall accuracy of 74.44% (Figure 4E).

The same diagnostic cutoff was then applied to the independent validation cohort to assess reproducibility. Consistent with the discovery cohort, OPLS‐DA based on hexanal showed separation between MPE and BPE samples in the validation cohort (Figure 4F). Hexanal levels remained significantly elevated in MPE compared with BPE (*Q* < 0.01; Figure 4G), and the dashed line again indicates the predefined diagnostic cutoff. ROC analysis in the validation cohort showed an AUC of 0.8339. Using the same cutoff of 4.5 × 10^6^, hexanal achieved a sensitivity of 75.00%, specificity of 65.71%, and accuracy of 70.15% (Figure 4H). To confirm the chemical identity of the detected compound, qualitative validation was performed using an authentic hexanal standard. The experimental sample and authentic standard showed identical retention time at 10.35 min and highly consistent characteristic fragment ions, including *m*/*z* 56, 57, 72, and 82 (Figure S3).

To place the diagnostic value of hexanal in a clinical context, serum CEA and pleural effusion CEA were evaluated in parallel. The comparative diagnostic performance of hexanal, pleural effusion CEA, and serum CEA in both cohorts is summarized in Table S5, and the corresponding ROC curves are shown in Figure S4. In the discovery cohort, hexanal showed a sensitivity of 70.30%, specificity of 78.30%, and accuracy of 74.40%. In the validation cohort, hexanal showed a sensitivity of 75.00%, specificity of 65.71%, and accuracy of 70.15%. Compared with serum CEA, hexanal showed higher sensitivity in both cohorts, whereas pleural effusion CEA showed higher specificity.

Overall, these results identify hexanal as the only VOC consistently altered in both LC‐associated MPE and tumor tissues. Its reproducible elevation in MPE, acceptable analytical precision, chemical confirmation using an authentic standard, and diagnostic performance in an independent validation cohort support its potential value as a pleural effusion‐based metabolic biomarker for LC‐associated MPE.

### 2.4 Investigation of the Generation Mechanism of Candidate Metabolic VOC Biomarkers

The purpose of this analysis was to explore the possible biological mechanism underlying hexanal accumulation in LC‐associated MPE and tumor tissues. Because hexanal is commonly regarded as an LPO‐related aldehyde, we investigated whether fatty acid metabolism and related oxidative metabolic pathways were altered in LC tumor cells using single‐cell transcriptomic data.

**FIGURE 5:**
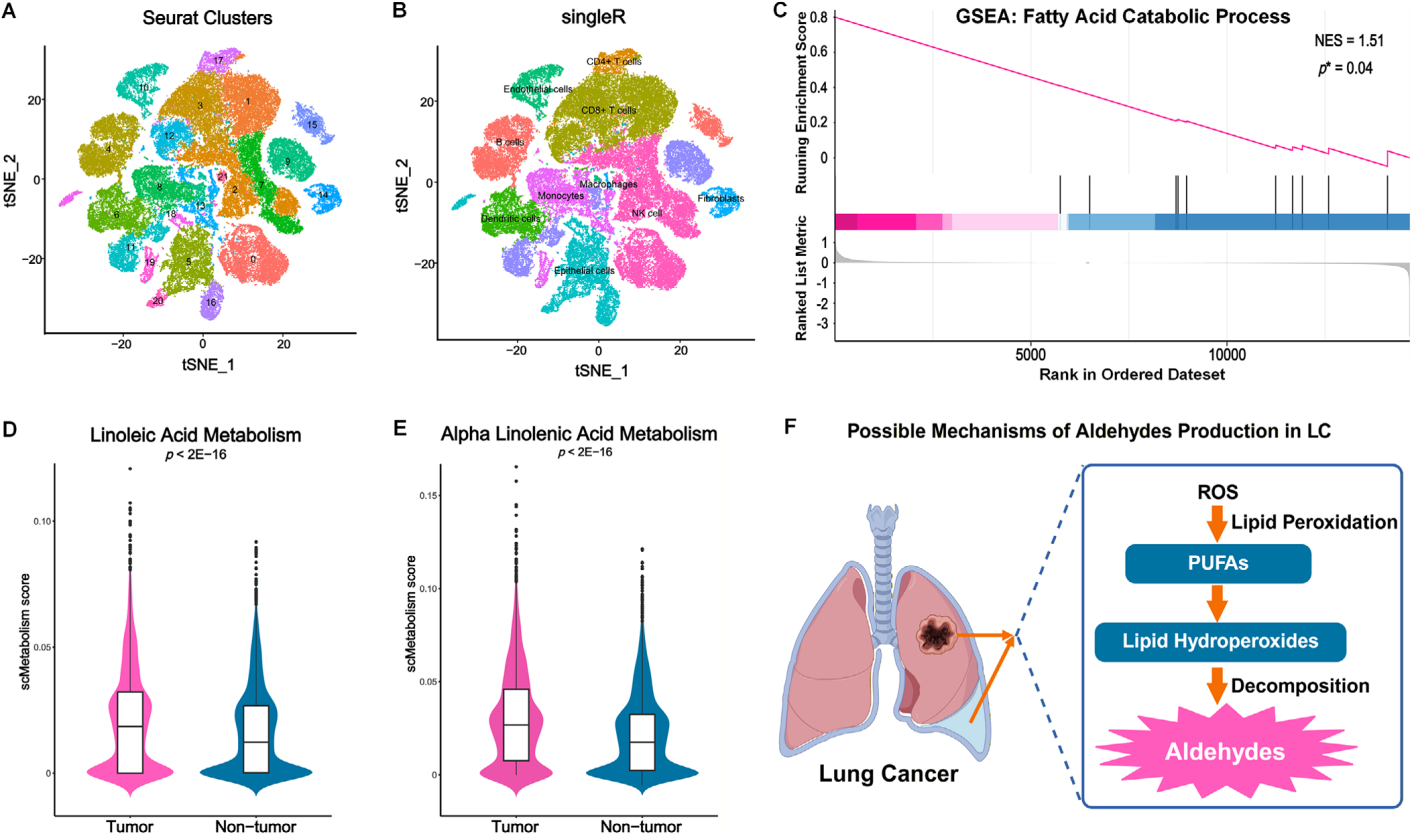
Single‐cell transcriptomic analysis and proposed mechanism of aldehyde production in LC. (A) t‐SNE plot of Seurat‐based clustering. (B) Cell type annotation by SingleR. (C) GSEA plot showing enrichment of the fatty acid catabolic process in LC tissues. (D and E) Violin plots showing the metabolic activity scores of linoleic acid metabolism and alpha‐linolenic acid metabolism in tumor and adjacent nontumor tissues. (F) Schematic illustration of the proposed mechanism of aldehyde production in LC. *Abbreviations*: GSEA, Gene Set Enrichment Analysis; LC, lung cancer; NES, normalized enrichment score; PUFAs, polyunsaturated fatty acids; ROS, reactive oxygen species; t‐SNE, t‐distributed stochastic neighbor embedding.

A publicly available single‐cell RNA sequencing dataset of LC tissues, GSE189357, was obtained from the Gene Expression Omnibus (GEO) database. After quality control, 79,073 high‐quality cells were retained for downstream analysis. Canonical correlation analysis (CCA)‐based integration followed by Seurat clustering identified 22 cell subclusters, as shown in the dimensional reduction plot (Figure 5A). Cell‐type annotation using SingleR classified these cells into 10 major cell types (Figure 5B). To distinguish malignant epithelial cells from nonmalignant epithelial cells, inferCNV analysis was performed, identifying 5500 malignant epithelial cells and 3660 normal control cells.

Pathway‐level analyses were then conducted to examine metabolic differences between malignant and normal epithelial cells. Gene Set Enrichment Analysis (GSEA) showed significant enrichment of the fatty acid catabolic process in tumor cells, with a normalized enrichment score of 1.51 and *p* = 0.04 (Figure 5C). Single‐cell metabolic pathway activity analysis further demonstrated marked activation of linoleic acid metabolism and alpha‐linolenic acid metabolism in tumor tissues, both reaching strong statistical significance (*p* ≤ 2 × 10^−^^1^ ^6^; Figure 5D,E). These two pathways are closely related to PUFA metabolism and may provide upstream substrates for LPO‐derived aldehyde generation.

Based on the integrated metabolomic and single‐cell transcriptomic findings, we propose a mechanistic hypothesis in which abnormal activation of fatty acid metabolism in LC promotes reactive oxygen species (ROS)‐associated LPO of PUFAs, thereby contributing to the accumulation of hexanal in the local tumor microenvironment and its enrichment in MPE. This proposed mechanism is summarized schematically in Figure 5F.

Collectively, these results suggest that hexanal accumulation in LC‐associated MPE may be linked to tumor fatty acid metabolic reprogramming and LPO. Although this analysis provides biological plausibility for hexanal as an LC‐associated VOC biomarker, direct experimental validation of its cellular origin and metabolic production pathway will be required in future studies.

## 3 Discussion

At present, biomarker research in LC gas biopsy has predominantly focused on single systemic biological matrices, such as exhaled breath, peripheral blood, or urine [18]. Although these samples are readily accessible, their metabolic profiles are highly susceptible to whole‐body circulatory regulation, dietary factors, and environmental exposure, which may compromise the specificity of metabolic VOCs for primary LC lesions [19]. In contrast, MPE, as a locoregional biofluid, is anatomically and biologically closely associated with pleural metastasis and tumor–microenvironment interactions, thereby providing a more proximal and tumor‐relevant metabolic context [20]. Based on these considerations, the present study selected MPE as the primary analytical matrix, with the aim of enhancing the specificity of LC‐associated VOC biomarkers.

In this study, 107 patients with LC‐associated MPE and 112 patients with BPE were enrolled, constituting one of the larger cohorts reported to date in pleural effusion‐based metabolomic investigations. The expanded sample size substantially improved statistical power and strengthened the robustness of the conclusions. More importantly, to our knowledge, this is the first study to systematically integrate VOC metabolic profiles derived from MPE with those obtained from LC tumor tissues, thereby establishing a cross‐biological matrix validation strategy. This approach facilitates the identification of candidate biomarkers that not only discriminate malignant from BPEs but also originate from tumor‐associated metabolic abnormalities and are stably enriched in pleural effusion. Through this strategy, hexanal was identified as the only VOC consistently shared between MPE and tumor tissues. Its diagnostic performance yielded AUC values of 0.7828 in the discovery cohort and 0.8339 in the independent validation cohort, indicating moderate‐to‐good discriminatory ability and satisfactory reproducibility. These findings suggest that hexanal represents a relatively stable LC‐associated metabolic signal across different biological matrices and is closely linked to tumor metabolism.

Previous studies have reported hexanal as a potential LC‐associated VOC across multiple biological samples [21]. Early breath analysis studies demonstrated significantly elevated hexanal levels in LC patients compared with healthy controls [22], while subsequent blood‐ and urine‐based investigations further confirmed its systemic elevation in cancer patients [23, 24]. Notably, tissue‐level metabolomic studies have consistently shown increased hexanal abundance in LC tissues relative to normal lung tissues [25]. Extending these observations, the present study demonstrates that hexanal is not only elevated in LC tumor tissues but is also significantly enriched in MPE compared with BPE, thereby establishing a potential biological link between tumor metabolic dysregulation and the VOC composition of pleural effusion.

Single‐cell transcriptomic analysis revealed significant activation of fatty acid metabolism‐related pathways in LC tissues, indicating pronounced metabolic reprogramming. These findings provide indirect evidence supporting an association between hexanal accumulation and LPO processes within the tumor microenvironment [26]. Under conditions of oxidative stress, PUFAs can generate medium‐chain aldehydes, including hexanal, through oxidative cleavage of ω‐6/ω‐7 fatty acids [7, 27]. Nevertheless, the present study does not provide direct experimental evidence for the cellular origin of hexanal. Future investigations incorporating in vitro LC cell models, oxidative stress modulation, and isotope‐labeled substrate tracing are warranted to definitively elucidate the metabolic pathways and cellular sources underlying hexanal production.

Several limitations of this study should be acknowledged. First, all MPE samples were derived from LC patients, whereas MPE can also occur in other malignancies, including breast, ovarian, and gastrointestinal cancers. Consequently, it remains unclear whether hexanal exhibits LC‐specific metabolic enrichment or represents a shared metabolic phenotype across multiple tumor types. Second, all MPE samples were associated with non‐small cell LC, and tumor tissue samples were restricted to lung adenocarcinoma. Given the well‐recognized heterogeneity of metabolic reprogramming across LC subtypes, these factors may limit the generalizability of the present findings to other histological subtypes, such as squamous cell carcinoma or small cell LC. Broader histological coverage will therefore be required in future studies.

During the untargeted VOC metabolomic analysis, several additional compounds were detected, including propofol, methyl diethyldithiocarbamate, toluene, and nonane. These VOCs are generally considered to originate predominantly from exogenous sources, such as anesthetic agents, therapeutic medications, plastic‐related emissions, or environmental contamination, rather than endogenous tumor metabolism [28, 29, 30]. Notably, none of these compounds were retained in the overlap between MPE‐specific VOCs and LC tissue‐specific VOCs, further underscoring the value of cross‐biological matrix validation in reducing exogenous interference and enhancing biomarker specificity.

Although hexanal demonstrated good diagnostic performance and offers advantages such as minimal invasiveness and the ability to reflect tumor metabolic status when detected in pleural effusion, the diagnostic utility of a single metabolite remains inherently limited. Moreover, aldehyde compounds are susceptible to exogenous contamination, oxidative artifacts during sample storage, and environmental VOC exposure, all of which may introduce bias into VOC‐based analyses. Although standardized sample handling procedures, strict low‐temperature storage conditions, and consistent analytical parameters were implemented to mitigate these effects, residual confounding cannot be entirely excluded. Future studies incorporating isotope‐labeled internal standards, procedural blanks, and longitudinal stability assessments will further strengthen methodological rigor. From a clinical perspective, hexanal may be more appropriately positioned as an adjunctive or complementary metabolic indicator, integrated with cytological examination, imaging modalities, and conventional tumor markers, rather than as an independent diagnostic biomarker.

In summary, this study demonstrates that the VOC metabolic profile of MPE reflects LC‐associated metabolic reprogramming and provides a tumor–proximal window into localized metabolic abnormalities. A diagnostic strategy based on VOC analysis of pleural effusion partially overcomes the limitations of conventional LC diagnostic approaches and lays the foundation for the development of low‐cost, minimally invasive, and highly specific diagnostic tools for LC.

## 4 Materials and Methods

### 4.1 Cohorts and Sample Collection

*Pleural Effusion Cohort*: From April 2021 to July 2024, this study enrolled 107 patients with LC‐associated MPE and 112 patients with BPE at the Departments of Respiratory Medicine and Infectious Diseases, The First Affiliated Hospital of the University of Science and Technology of China. The screening process is shown in Figure S5A. *Tissue Cohort*: From April 2021 to July 2023, the study included 14 patients who underwent radical surgery for LC at the Department of Thoracic Surgery of the First Affiliated Hospital of the University of Science and Technology of China. The sample selection process is illustrated in Figure S5B. The inclusion criteria for subjects are detailed in Supporting Information: Methods. Clinical characteristics of the patients are provided in Table 3. This study was approved by the Ethics Committee of the First Affiliated Hospital of the University of Science and Technology of China (Ethics Approval Number: 2021 KY‐059). Each study participant provided informed consent.

**TABLE 3:** Clinical characteristics of patients in the pleural effusion and tissue cohorts.

	Pleural effusion cohort (*n* = 219)				
	Discovery cohort (*n* = 153)		Validation cohort (*n* = 66)		Tissue cohort
Variables	MPE (*n* = 75)	BPE (*n* = 78)	MPE (*n* = 32)	BPE (*n* = 34)	(*n* = 14)
**Gender**					
Male	35 (46.7%)	37 (47.4%)	14 (43.8%)	18 (52.9%)	9 (64.3%)
Female	40 (53.3%)	41 (52.6%)	18 (56.2%)	16 (47.1%)	5 (35.7%)
**Age, years**	61.5 ± 10.5	59.5 ± 9.7	64.4 ± 10.6	62.2 ± 13.5	60.1 ± 9.7
(mean ± SD)					
**Histological** **classification of LC**					
Adenocarcinoma	65 (86.7%)	—	30 (93.8%)	—	14 (100.0%)
Squamous cell carcinoma	10 (13.3%)	—	2 (6.2%)	—	0 (0%)
SCLC	0 (0%)	—	0 (0%)	—	0 (0%)
**No prior antitumor therapy**	75 (100.0%)	—	32 (100.0%)	—	14 (100.0%)
**No metabolic diseases**	75 (100.0%)	78 (100.0%)	32 (100.0%)	34 (100.0%)	14 (100.0%)
**BPE etiology**					—
Tuberculous pleurisy	—	45 (57.7%)	—	20 (58.8%)	—
Pneumonia	—	33 (42.3%)	—	14 (41.2%)	—
**No antibiotic or steroid use within 1 month**	75 (100.0%)	78 (100.0%)	32 (100.0%)	34 (100.0%)	—
**Appearance of pleural effusion**					—
Watery/serous	48 (64.0%)	58 (74.4%)	20 (62.5%)	25 (73.5%)	—
Bloody/blood‐tinged	26 (34.7%)	5 (6.4%)	12 (37.5%)	1 (2.9%)	—
Purulent/turbid	1 (1.3%)	15 (19.2%)	0 (0%)	8 (23.5%)	—
**Smoking history**					
Never smoker	51 (68.0%)	49 (62.8%)	17 (53.1%)	19 (55.9%)	12 (85.7%)
Former smoker	10 (13.3%)	9 (11.5%)	8 (25.0%)	5 (14.7%)	2 (14.3%)
Current smoker	14 (18.7%)	20 (25.6%)	7 (21.9%)	10 (29.4%)	0 (0%)

Abbreviations: BPE, benign pleural effusion; MPE, malignant pleural effusion; LC, lung cancer; SCLC, small cell lung cancer; SD, standard deviation; —, not applicable.

The inclusion and exclusion criteria, sample preparation, detection procedures, and data preprocessing parameters are provided in Supporting Information: Methods. To assess the generalization performance of the model, the 219 enrolled pleural effusion samples (107 from the MPE group and 112 from the BPE group) were randomly divided into discovery and validation cohorts at a ratio of 7:3 [31]. Specifically, the discovery cohort comprised 153 samples (MPE 75, BPE 78), while the validation cohort comprised 66 samples (MPE 32, BPE 34).

### 4.2 Sample Detection and Data Preprocessing

Metabolomics analysis of pleural effusion and tissue samples was conducted using a gas chromatography–triple quadrupole mass spectrometer (TSQ Quantum XLS; Thermo Fisher Scientific, USA), with additional details provided in Supporting Information: Methods. The HS‐SPME‐GC–MS conditions used in this study, including fiber type, extraction temperature, extraction time, and desorption procedure, were based on our previously validated method [32, 33]. Sample input amounts were further optimized in the present study. As shown in Figure S6, 1.0 mL pleural effusion and 0.10 g tissue were selected for subsequent analysis. To monitor system suitability throughout the GC–MS analytical period, the TIC signal of the perfluorotributylamine (PFTBA) standard was injected once per analytical day. The instrument exhibited acceptable stability, with an overall RSD of 18.51% for the PFTBA TIC across all analytical days, consistent with commonly accepted quality control criteria in GC–MS metabolomics (<30% RSD) (Figure S7) [33, 34, 35, 36]. For the preprocessing of GC–MS raw data, this study utilized the XCMS Online platform (https://xcmsonline.scripps.edu/). The preprocessing workflow encompassed peak detection/extraction, noise reduction, deconvolution, and alignment to ensure the reliability of the data (further specifics available in Supporting Information: Methods) [37, 38, 39].

### 4.3 Differential Metabolite Selection Strategy

A combined univariate and multivariate analytical strategy was applied to identify differential metabolites. First, all characteristic ions were subjected to the Mann–Whitney *U* test, and the resulting *p* values were adjusted for multiple comparisons using the Benjamini–Hochberg FDR procedure. Features with *q* values < 0.05 were considered statistically significant [40]. FC values were calculated, and considering that the RSD of instrumental fluctuation was 18.51%, a conservative FC threshold of >2.0 or <0.5 (|log_2_FC| > 1) was applied to minimize the likelihood that observed differences arose from instrumental variability rather than true biological variation [41]. In parallel, OPLS‐DA with UV scaling was performed, model robustness was evaluated by 200 permutation tests, and VIP values were calculated [42]. Only characteristic ions simultaneously meeting the criteria of *q* < 0.05, |log_2_FC| > 1, and VIP > 1.5 were defined as differential characteristic ions.

Subsequently, differential characteristic ions were annotated as putative VOCs based on the NIST mass spectral library in combination with RI information [41]. The peak areas of candidate VOCs were compared between MPE and BPE, as well as between tumor and adjacent nontumor tissues, using the Mann–Whitney *U* test, followed by FDR correction. VOCs with *Q* values < 0.05 were defined as differential VOCs. Finally, differential VOCs identified in the pleural effusion discovery cohort were intersected with those identified in the tissue cohort, and only VOCs consistently differential in both datasets were retained as candidate LC‐associated metabolites. Qualitative confirmation was subsequently performed using authentic chemical standards by matching retention times, characteristic ions, and mass spectral fragmentation patterns. ROC curve analysis was applied to evaluate the diagnostic efficiency of the metabolites, calculating sensitivity, specificity, and optimal cutoff values [43].

### 4.4 Preparation of Standard Curve Solutions

The chemical standards used in the experiments included hexanal (purity ≥ 99.0%), methanol (purity ≥  99.9%), and benzaldehyde (purity ≥ 99.5%, used as an internal standard), all purchased from Shanghai Aladdin Biochemical Technology Co., Ltd. The hexanal stock solution (500 µg/mL) and the internal standard stock solution (1 mg/mL) were prepared using methanol as the solvent and stored at −80°C. In the standard curve experiments, thawed hexanal and internal standard stock solutions were further diluted with methanol to prepare the hexanal working solution (10 µg/mL) and the internal standard working solution (1 µg/mL) [24].

### 4.5 Single‐Cell Sequencing Data Analysis

This study analyzed the single‐cell transcriptomic dataset GSE189357 for LC obtained from the GEO database, which includes nine samples [44]. The data analysis workflow was as follows: first, the Seurat package was used for quality control, applying the filters: minGene > 200, maxGene < 2000, and mitochondrial gene percentage < 15%. The cells that passed quality control were normalized and converted into Seurat objects, and the top 2000 highly variable genes were identified. CCA was performed for cross‐sample integration [45]. The FindIntegrationAnchors function (dims = 1:20) was used to identify anchors, and data integration was completed using the IntegrateData function. Subsequently, the integrated expression matrix was normalized and scaled, and the top 20 principal components were extracted based on principal component analysis. Unsupervised clustering was conducted using the Shared Nearest Neighbor algorithm with a resolution of 0.5. To exclude multiplet capture events, the DoubletFinder package was employed to calculate doublet probabilities. Cell annotation for immune cell populations was performed using the SingleR package against a nonmalignant reference set, while malignant epithelial cells were characterized using the copy number variation (CNV) scores calculated by the inferCNV package [46]. Next, normalized data from benign and malignant epithelial cell populations were extracted, and differential gene analysis was conducted using the limma package. GSEA was performed using the clusterProfiler package, with *p* < 0.05 indicating significant pathway enrichment [47]. Finally, using the scMetabolism package and Kyoto Encyclopedia of Genes and Genomes database‐defined metabolic pathway gene sets, the AUCell algorithm was employed to assess single‐cell metabolic pathway activity, and gene set enrichment scores were used to quantify the spatial heterogeneity of metabolic states [48].

## Author Contributions

Hongzhi Wang conceived and supervised the study and served as the guarantor of the manuscript. Wenting Liu, Yannan Chu, Dianlong Ge, and Hongzhi Wang contributed to study design, data analysis, and data interpretation. Li Ke, Xiangxue Zheng, Jijuan Zhou, and Yan Lu contributed to study design and data collection. Jian Qi, Yajing Chu, and Yue Liu contributed to data analysis and interpretation. All authors contributed to manuscript drafting and revision. Dianlong Ge, Yannan Chu, and Hongzhi Wang verified the underlying data. All authors had full access to the data and take responsibility for the integrity of the data and the accuracy of the data analysis. All authors have read and approved the final manuscript.

## Funding

This work was supported by the National Natural Science Foundation of China (No. 21876176) and the President Foundation of Hefei Institutes of Physical Science (No. YZJJZX202009).

## Conflicts of Interest

The authors declare no conflicts of interest.

## Ethics Statement

This study was approved by the Ethics Committee of the First Affiliated Hospital of the University of Science and Technology of China (Approval No. 2021 KY‐059). Written informed consent was obtained from all participants.

## Supporting information




**Table S1**: Differential characteristic ions in the pleural effusion discovery cohort (*q* < 0.05, |log_2_FC| > 1, and VIP > 1.5) **Table S2**: Differential characteristic ions in the tumor tissue cohort (*q* < 0.05, |log_2_FC| > 1, and VIP > 1.5) **Table S3**: Intraday precision of hexanal at different concentration levels **Table S4**: Interday precision of hexanal at different concentration levels **Table S5**: Diagnostic performance of serum CEA, PE CEA, and hexanal for distinguishing LC–associated MPE **Figure S1**: Storage‐time stability of hexanal in aliquoted pleural effusion samples stored at −80°C **Figure S2**: Intraday and interday precision of hexanal measured by HS‐SPME‐GC–MS **Figure S3**: Qualitative validation of hexanal in pleural effusion by GC–MS using an authentic chemical standard **Figure S4**: ROC curves of serum CEA, pleural effusion CEA, and hexanal for distinguishing LC‐associated MPE from BPE in the discovery and validation cohorts **Figure S5**: Patient enrollment process for the pleural effusion and tissue cohorts **Figure S6**: Optimization of sample input amounts for HS‐SPME‐GC–MS analysis **Figure S7**: Daily PFTBA TIC signal intensity during the GC–MS analytical period

## Data Availability

The original data supporting the findings of this study are available from the corresponding author upon reasonable request.
