# Supplementary material for: Gas Biopsy of Pleural Effusion to Diagnose Lung Cancer by Mass Spectrometry
Source: MedComm (2020). 2026 Jun 4;7(6):e70787. doi: 10.1002/mco2.70787 (PMC13238847; doi:10.1002/mco2.70787)
Supplement: Supplementary file 1 — Table S1: Differential characteristic ions in the pleural effusion discovery cohort (q < 0.05, |log2FC| > 1, and VIP > 1.5) Table S2: Differential characteristic ions in the tumor tissue cohort (q < 0.05, |log2FC| > 1, and VIP > 1.5) Table S3: Intraday precision of hexanal at different concentration levels Table S4: Interday precision of hexanal at different concentration levels Table S5: Diagnostic performance of serum CEA, PE CEA, and hexanal for distinguishing LC–associated MPE Figure S1: Storage‐time stability of hexanal in aliquoted pleural effusion samples stored at −80°C Figure S2: Intraday and interday precision of hexanal measured by HS‐SPME‐GC–MS Figure S3: Qualitative validation of hexanal in pleural effusion by GC–MS using an authentic chemical standard Figure S4: ROC curves of serum CEA, pleural effusion CEA, and hexanal for distinguishing LC‐associated MPE from BPE in the discovery and validation cohorts Figure S5: Patient enrollment process for the pleural effusion and tissue cohorts Figure S6: Optimization of sample input amounts for HS‐SPME‐GC–MS analysis Figure S7: Daily PFTBA TIC signal intensity during the GC–MS analytical period [file MCO2-7-e70787-s001.docx]

**Supplementary Information**

**Gas Biopsy of Pleural Effusion to Diagnose Lung Cancer by Mass Spectrometry**

Wenting Liu^1,2,3,4^, Jian Qi^1,2^, Yajing Chu^5^, Jijuan Zhou^1,2^, Yue Liu^1,2,4^, Li Ke^1,2,4,6^, Xiangxue Zheng^1,2,4^, Yan Lu^1,2,4^, Dianlong Ge^1,2,4,*^, Yannan Chu^1,2,4,*^, Hongzhi Wang^1,2,*^

^1^Hefei Cancer Hospital of CAS, Institute of Health and Medical Technology,

Hefei Institutes of Physical Science, Chinese Academy of Sciences (CAS), Hefei,

Anhui 230031, China.

^2^Science Island Branch, Graduate School of the University of Science and Technology of China, Hefei, Anhui 230026, China.

^3^Department of Geriatric Medicine, The First Affiliated Hospital of the University of Science and Technology of China, Division of Life Sciences and Medicine, University of Science and Technology of China, Hefei, Anhui 230001, China.

^4^Anhui Province Key Laboratory of Medical Physics and Technology, Hefei, Anhui 230031, China.

^5^Key Laboratory of Biodiversity Conservation and Characteristic Resource Utilization in Southwest Anhui, School of Life Sciences, Anqing Forestry Technology Innovation Research Institute, Anqing Normal University, Anhui 246000, China.

^6^Department of Thoracic Surgery, The First Affiliated Hospital of the University of Science and Technology of China, Division of Life Sciences and Medicine, University of Science and Technology of China, Hefei, Anhui 230001, China.

**Corresponding authors:**

**Dianlong Ge, PhD**

Institute of Health and Medical Technology, Hefei Institutes of Physical Science, Chinese Academy of Sciences, Hefei, 230031, Anhui, China.

E-mail: [gedl@cmpt.ac.cn](mailto:gedl@cmpt.ac.cn)

**Yannan Chu, PhD**

Institute of Health and Medical Technology, Hefei Institutes of Physical Science, Chinese Academy of Sciences, Hefei, 230031, Anhui, China.

E-mail: [ychu@aiofm.ac.cn](mailto:ychu@aiofm.ac.cn)

**Hongzhi Wang, PhD**

Institute of Health and Medical Technology, Hefei Institutes of Physical Science, Chinese Academy of Sciences, Hefei, 230031, Anhui, China.

E-mail: [wanghz@hfcas.ac.cn](mailto:wanghz@hfcas.ac.cn)

**Table of contents**

**[Supplementary Methods](#_Toc851) 1**

[Inclusion criteria and exclusion criteria for participants](#_Toc17597) 1

[Sample detection](#_Toc25708) 3

[Data preprocessing.................................................................................................................](#_Toc562)4

[References](#_Toc18979) 5

**[Supplementary Tables](#_Toc14458) 6**

Table S1: Differential characteristic ions in the pleural effusion discovery cohort (*q* < 0.05, |log₂FC| > 1, and VIP > 1.5)...................................................................................................6

Table S2: Differential characteristic ions in the tumor tissue cohort (*q* < 0.05, |log₂FC| > 1, and VIP > 1.5)........................................................................................................................8

Table S3: Intra-day precision of hexanal at different concentration levels..........................10

[Table S4: Inter-day precision of hexanal at different concentration levels..........................](#_Toc23843)11

[Table S5: Diagnostic performance of serum CEA, PE CEA, and hexanal for distinguishing LC–associated MPE.............................................................................................................1](#_Toc6537)2

**[Supplementary F](#_Toc14458)igures..........................................................................................................13**

Figure S1: Storage-time stability of hexanal in aliquoted pleural effusion samples stored at −80°C....................................................................................................................................13

[Figure S2: Intra-day and inter-day precision of hexanal measured by HS-SPME-GC–MS...............................................................................................................](#_Toc14563)14

Figure S3: Qualitative validation of hexanal in pleural effusion by GC–MS using an authentic chemical standard..................................................................................................15

Figure S4: ROC curves of serum CEA, pleural effusion CEA, and hexanal for distinguishing LC-associated MPE from BPE in the discovery and validation cohorts..................................................................................................................................16

[Figure S5: Patient enrollment process for the pleural effusion and tissue cohorts...](#_Toc8737) 17

[Figure S6: Optimization of sample input amounts for HS-SPME-GC–MS analysis...........](#_Toc438)18

Figure S7: Daily PFTBA TIC signal intensity during the GC–MS analytical period[..........](#_Toc32119)19

**Supplementary Methods**

**Inclusion and exclusion criteria for participants:**

Malignant Pleural Effusion (MPE) Cohort

Inclusion criteria:

1. Age ≥ 18 years.
2. No prior anti-tumor therapy, including surgery, radiotherapy, chemotherapy, targeted therapy, or immunotherapy.
3. Pleural effusion fulfilling Light’s criteria for exudates.
4. Cytological or histopathological confirmation of lung cancer cells in pleural effusion obtained via thoracentesis.
5. Ability to safely undergo thoracentesis.

Exclusion criteria:

1. Incomplete clinical information or undetermined etiology of pleural effusion.
2. History or concurrent diagnosis of other malignancies.
3. Use of antibiotics or corticosteroids within the past 30 days.
4. Indwelling pleural catheter in place for > 48 hours before sampling.
5. Presence of metabolic diseases, including diabetes, hypertension, thyroid disorders, or other endocrine diseases.
6. Inability to tolerate thoracentesis.

Benign Pleural Effusion (BPE) Cohort

Inclusion criteria:

1. Age ≥ 18 years.
2. Pleural effusion fulfilling Light’s criteria for exudates.
3. Negative cytological examination for malignant cells.
4. Definitive benign diagnosis established by:
5. Radiological or clinical evidence of pneumonia with resolution after anti-inflammatory treatment.
6. Tuberculous pleurisy confirmed by clinical, microbiological, or immunological criteria.
7. A minimum 3-month follow-up showing complete or partial absorption of effusion without tumor occurrence.
8. Ability to safely undergo thoracentesis.

Exclusion criteria:

1. Incomplete clinical information or uncertain etiology.
2. Use of antibiotics or corticosteroids within the past 30 days (unless they were used for treatment of the benign disease and the sample was collected before therapy).
3. Presence of metabolic diseases, including diabetes, hypertension, thyroid disorders, etc.
4. Inability to tolerate thoracentesis.
5. Indwelling pleural drainage catheter for > 48 h prior to sampling.

Lung Cancer Tissue Cohort

Inclusion criteria:

1. Age ≥ 18 years.
2. Underwent radical surgical resection for primary lung cancer.
3. Postoperative histopathological confirmation of lung cancer and matched adjacent non-tumor lung tissue.

Exclusion criteria:

1. History or presence of other malignancies.
2. Presence of metabolic diseases (diabetes, hypertension, thyroid disorders, etc.).
3. Non-matching pathology results (e.g., unexpected benign lesions).

**Sample detection**

First, a 65 μm PDMS/DVB-coated SPME fiber was placed in the GC injection port and conditioned at 200°C for 30 minutes to remove potential impurities^1^. Subsequently, 5 mL of pleural effusion sample was retrieved from the -80°C freezer, allowed to thaw at room temperature, and then 1 mL was transferred to a 15 mL glass extraction vial. The extraction vial was placed in a 37°C incubator for 10 minutes, after which the SPME fiber was inserted for HS extraction for 40 minutes. Once completed, the fiber was immediately withdrawn^1,2^. Finally, the SPME fiber was quickly inserted into the vaporization chamber of the mass spectrometer, where it was held for 30 seconds to facilitate desorption before being withdrawn^1^. The same procedure was applied for tissue samples: a 0.1 g tissue sample was thawed and placed in a 15 mL glass extraction vial, followed by the same VOC extraction and desorption process.

The analysis was performed using a GC-triple quadrupole mass spectrometry system (TSQ Quantum XLS; Thermo Fisher Scientific, USA). The chromatography column was a TG-624SILMS capillary column (30 m × 0.32 mm × 1.80 μm; Thermo Scientific, Bellefonte, USA), with high-purity helium (purity ≥ 99.999%) as the carrier gas at a flow rate of 1.5 mL/min. The injection port temperature was set at 200°C, using a splitless injection mode. The column temperature program was as follows: an initial temperature of 40°C held for 1 minute, then ramped at 5°C/min to 180°C and held for 2 minutes. The transfer line and ion source temperatures were maintained at 200°C. MS analysis was conducted in full scan mode with a mass scanning range of 45-300 amu, and electron impact ionization energy was set at 70 eV^3^.

**Data preprocessing**

To reduce experimental drift caused by factors such as column degradation and temperature fluctuations, this study utilized XCMS Online (https://xcmsonline.scripps.edu/) to preprocess raw GC–MS data.^4^ The preprocessing workflow included peak alignment, noise reduction, deconvolution, and peak extraction to minimize nonlinear bias and ensure data reliability. The specific steps were as follows: first, the RAW format raw data was converted to mzML format using ProteoWizard 3.0.22015^5^. Next, parameters were set in XCMS Online: the instrument type was selected as "GC/Single Quad (matchedFilter)", the chromatography separation type was set to "GC-EI", and the mass spectrometry type was specified as "single quadrupole MS". Peak detection was performed using the "matchedFilter" algorithm (FWHM = 3, step = 0.25, S/N = 10) to enhance sensitivity. Retention time correction employed the "obiwarp" algorithm (profStep = 0.25) to reduce nonlinear drift. Grouping parameters were set to mzwid = 0.25, minfrac = 0.5, and bw = 3 to ensure precise mass matching^6^.

**References**

1 Liu Y, Ge D, Zhou J, et al. HS–SPME–GC–MS Untargeted Analysis of Normal Rat Organs Ex Vivo: Differential VOC Discrimination and Fingerprint VOC Identification. Analytical chemistry (Washington) 2023; 95:11375-11382.

2 Ge D, Zhou J, Chu Y, et al. Distinguish oral-source VOCs and control their potential impact on breath biomarkers. Anal Bioanal Chem 2022; 414:2275-2284.

3 Chu Y, Ge D, Zhou J, et al. Controlling glycolysis to generate characteristic volatile organic compounds of lung cancer cells. Sci Rep 2024; 14:16561.

4 Tautenhahn R, Patti GJ, Rinehart D, et al. XCMS Online: A Web-Based Platform to Process Untargeted Metabolomic Data. Anal Chem 2012; 84:5035-5039.

5 Kessner D, Chambers M, Burke R, et al. ProteoWizard: open source software for rapid proteomics tools development. Bioinformatics 2008; 24:2534-2536.

6 Forsberg EM, Huan T, Rinehart D, et al. Data processing, multi-omic pathway mapping, and metabolite activity analysis using XCMS Online. Nat Protoc 2018; 13:633-651.

**Supplementary Tables**

Table S1: Differential characteristic ions in the pleural effusion discovery cohort (*q* < 0.05, |log₂FC| > 1, and VIP > 1.5).

| Characteristic | *q* value | log_2_(FC) | VIP | RT | VOC | CAS | RSI |
| --- | --- | --- | --- | --- | --- | --- | --- |
| ion |  |  |  | (min) |  |  |  |
| M45T2 | 2.63E-07 | 3.52 | 2.03 | 2.22 | N/A | | |
| M46T2 | 7.34E-07 | 3.54 | 2.08 |  |  |  |  |
| M47T2 | 3.31E-06 | 4.41 | 1.81 |  |  |  |  |
| M65T9 | 4.15E-07 | 1.11 | 3.08 | 8.73 | Toluene | 108-88-3 | 931 |
| M63T9_2 | 3.76E-06 | 1.09 | 2.96 |  |  |  |  |
| M90T9 | 1.11E-04 | 1.12 | 2.74 |  |  |  |  |
| M114T9 | 9.09E-04 | 1.49 | 1.63 | 9.08 | N/A | | |
| M72T10 | 0.04 | 1.01 | 1.66 | 10.35 | Hexanal | 66-25-1 | 824 |
| M133T11 | 2.11E-05 | 1.23 | 2.54 | 10.77 | Column bleeding | | |
| M149T11 | 2.93E-05 | 1.22 | 2.45 |  |  |  |  |
| M134T11 | 3.04E-05 | 1.30 | 2.47 |  |  |  |  |
| M150T11 | 4.61E-05 | 1.24 | 2.46 |  |  |  |  |
| M59T13 | 2.63E-07 | 1.14 | 2.73 | 12.56 | Nonane | 111-84-2 | 924 |
| M143T16 | 7.46E-03 | -1.64 | 1.52 | 15.98 | Decane | 124-18-5 | 934 |
| M142T16 | 0.02 | -1.62 | 1.53 |  |  |  |  |
| M223T17 | 2.99E-04 | 1.03 | 2.11 | 16.70 | Column bleeding | | |
| M226T17 | 3.88E-04 | 1.06 | 1.92 |  |  |  |  |
| M146T18 | 0.02 | 2.93 | 2.14 | 17.60 | 1,2-Dichlorobenzene | 95-50-1 | 909 |
| M148T18 | 0.02 | 2.92 | 2.14 |  |  |  |  |
| M150T18 | 0.02 | 2.97 | 2.17 |  |  |  |  |
| M120T20 | 2.85E-05 | -1.12 | 1.77 | 20.21 | Acetophenone | 98-86-2 | 918 |
| M51T20 | 3.26E-05 | -1.11 | 1.85 |  |  |  |  |
| M77T20 | 3.26E-05 | -1.10 | 1.81 |  |  |  |  |
| M105T20 | 3.38E-05 | -1.07 | 1.74 |  |  |  |  |
| M121T20 | 3.43E-05 | -1.16 | 1.84 |  |  |  |  |
| M78T20 | 3.72E-05 | -1.09 | 1.82 |  |  |  |  |
| M50T20 | 3.79E-05 | -1.13 | 1.89 |  |  |  |  |
| M74T20 | 4.56E-05 | -1.24 | 1.76 |  |  |  |  |
| M106T20 | 4.56E-05 | -1.08 | 1.77 |  |  |  |  |
| M52T20 | 4.90E-05 | -1.16 | 1.79 |  |  |  |  |
| M76T20 | 6.93E-05 | -1.11 | 1.83 |  |  |  |  |
| M300T20 | 9.09E-04 | -1.05 | 1.63 | 20.40 | N/A | | |
| M82T21 | 0.02 | 1.03 | 1.52 | 20.74 | Nonanal | 124-19-6 | 914 |
| M171T22_2 | 3.72E-05 | -1.99 | 1.86 | 22.43 | Dodecane | 112-40-3 | 929 |
| M86T22 | 5.87E-05 | -1.84 | 1.77 |  |  |  |  |
| M72T22 | 6.11E-05 | -1.87 | 1.77 |  |  |  |  |
| M71T22 | 1.12E-04 | -1.84 | 1.76 |  |  |  |  |
| M85T22 | 1.13E-04 | -1.85 | 1.77 |  |  |  |  |
| M113T22 | 1.93E-04 | -1.78 | 1.72 |  |  |  |  |
| M141T22_1 | 2.18E-04 | -1.86 | 1.78 |  |  |  |  |
| M84T22 | 3.49E-04 | -1.73 | 1.74 |  |  |  |  |
| M99T22_1 | 3.51E-04 | -1.84 | 1.75 |  |  |  |  |
| M112T22 | 3.84E-04 | -1.75 | 1.74 |  |  |  |  |
| M126T22 | 3.88E-04 | -1.77 | 1.77 |  |  |  |  |
| M56T22 | 4.03E-04 | -1.83 | 1.76 |  |  |  |  |
| M127T22 | 5.09E-04 | -1.77 | 1.73 |  |  |  |  |
| M70T22 | 5.81E-04 | -1.75 | 1.73 |  |  |  |  |
| M140T22_2 | 1.45E-03 | -1.61 | 1.70 |  |  |  |  |
| M172T22_2 | 3.26E-05 | -2.02 | 1.89 |  |  |  |  |
| M170T22 | 5.18E-05 | -1.96 | 1.86 |  |  |  |  |
| M57T22 | 5.33E-05 | -1.91 | 1.80 |  |  |  |  |
| M175T25 | 4.43E-08 | 2.03 | 2.34 | 24.55 | 1,2,2-  Tribromobutane | 3675-69-2 | 866 |
| M140T26 | 0.05 | -1.15 | 1.77 | 25.55 | Column bleeding | | |
| M141T26 | 6.81E-04 | -1.52 | 2.30 | 26.14 | N/A | | |
| M186T27 | 5.81E-04 | -1.33 | 1.86 | 26.55 | N/A | | |
| M188T27 | 0.02 | -1.32 | 1.61 | 26.55 | N/A | | |
| M199T28 | 0.02 | -1.44 | 1.67 | 28.13 | Tetradecane | 629-59-4 | 901 |

CAS, Chemical Abstracts Service; FC, fold change; N/A, not available; *q* value, false discovery rate (FDR)-adjusted *p* value; RSI, reverse search index; RT, retention time; VIP, variable importance in projection; VOC, volatile organic compound.

Table S2: Differential characteristic ions in the tumor tissue cohort (*q* < 0.05, |log₂FC| > 1, and VIP > 1.5).

| Characteristic | *q* value | log_2_(FC) | VIP | RT | VOC | CAS | RSI |
| --- | --- | --- | --- | --- | --- | --- | --- |
| ion |  |  |  | (min) |  |  |  |
| M64T3 | 0.04 | -2.11 | 1.82 | 2.75 | Carbon disulphide | 75-15-0 | 890 |
| M78T3 | 0.04 | -2.04 | 1.72 |  |  |  |  |
| M80T3 | 0.01 | -2.55 | 1.71 |  |  |  |  |
| M76T3 | 0.04 | -2.04 | 1.72 |  |  |  |  |
| M137T10 | 0.01 | 1.83 | 2.50 | 10.34 | Hexanal | 66-25-1 | 824 |
| M153T10 | 0.01 | 1.65 | 2.36 |  |  |  |  |
| M288T15_2 | 0.02 | 3.83 | 2.09 | 14.74 | N/A |  |  |
| M235T15 | 0.02 | 1.52 | 1.78 |  |  |  |  |
| M75T22 | 0.04 | -1.40 | 2.06 | 22.20 | N/A |  |  |
| M100T22 | 0.03 | -1.23 | 2.10 |  |  |  |  |
| M72T22 | 0.01 | -1.21 | 2.26 |  |  |  |  |
| M128T24 | 0.02 | -2.18 | 1.72 | 23.64 | N/A |  |  |
| M279T29 | 0.01 | 2.31 | 1.76 | 28.84 | Propofol | 2078-54-8 | 915 |
| M294T30_2 | 0.04 | -2.46 | 1.76 | 29.64 | N/A |  |  |
| M162T30 | 0.01 | -2.08 | 2.35 | 29.82 | Methyl diethyl- dithiocarbamate | 686-07-7 | 965 |
| M166T30 | 0.01 | -2.06 | 2.42 |  |  |  |  |
| M119T30 | 0.01 | -2.17 | 2.54 |  |  |  |  |
| M136T30 | 0.01 | -2.31 | 2.41 |  |  |  |  |
| M73T30_2 | 0.01 | -1.83 | 2.41 |  |  |  |  |
| M104T30 | 0.01 | -2.08 | 2.37 |  |  |  |  |
| M77T30 | 0.01 | -2.11 | 2.47 |  |  |  |  |
| M150T30 | 0.01 | -2.25 | 2.47 |  |  |  |  |
| M165T30 | 0.01 | -2.09 | 2.43 |  |  |  |  |
| M70T30 | 0.01 | -1.75 | 2.45 |  |  |  |  |
| M58T30 | 0.01 | -1.79 | 2.40 |  |  |  |  |
| M49T30 | 0.01 | -1.97 | 2.37 |  |  |  |  |
| M148T30_1 | 0.01 | -2.15 | 2.39 |  |  |  |  |
| M62T30 | 0.01 | -1.89 | 2.38 |  |  |  |  |
| M90T30 | 0.04 | -1.75 | 2.06 |  |  |  |  |
| M100T30 | 0.02 | -1.78 | 2.09 |  |  |  |  |
| M45T30 | 0.01 | -1.88 | 2.44 |  |  |  |  |
| M84T30 | 0.01 | -1.93 | 2.50 |  |  |  |  |
| M79T30 | 0.01 | -1.96 | 2.41 |  |  |  |  |
| M78T30 | 0.01 | -1.93 | 2.33 |  |  |  |  |
| M66T30 | 0.01 | -2.03 | 2.48 |  |  |  |  |
| M167T30 | 0.01 | -2.05 | 2.47 |  |  |  |  |
| M75T30 | 0.01 | -1.95 | 2.43 |  |  |  |  |
| M130T30 | 0.01 | -2.71 | 2.36 |  |  |  |  |
| M60T30 | 0.01 | -1.88 | 2.38 |  |  |  |  |
| M63T30 | 0.02 | -1.88 | 2.29 |  |  |  |  |
| M46T30 | 0.01 | -1.79 | 2.26 |  |  |  |  |
| M117T30 | 0.01 | -2.08 | 2.41 |  |  |  |  |
| M59T30 | 0.01 | -1.90 | 2.42 |  |  |  |  |
| M61T30 | 0.01 | -1.91 | 2.41 |  |  |  |  |
| M72T30 | 0.01 | -1.93 | 2.44 |  |  |  |  |
| M91T30 | 0.01 | -1.98 | 2.45 |  |  |  |  |
| M114T30 | 0.01 | -2.03 | 2.41 |  |  |  |  |
| M54T30 | 0.01 | -1.83 | 2.39 |  |  |  |  |
| M164T30 | 0.01 | -2.10 | 2.44 |  |  |  |  |
| M163T30 | 0.01 | -2.10 | 2.42 |  |  |  |  |
| M160T30 | 0.04 | -1.76 | 1.97 |  |  |  |  |
| M92T30 | 0.01 | -1.96 | 2.45 |  |  |  |  |
| M94T30 | 0.01 | -2.26 | 2.42 |  |  |  |  |
| M93T30 | 0.01 | -1.97 | 2.45 |  |  |  |  |
| M102T30 | 0.01 | -2.11 | 2.38 |  |  |  |  |
| M52T30 | 0.01 | -2.05 | 2.39 |  |  |  |  |
| M108T30 | 0.01 | -2.40 | 2.28 |  |  |  |  |
| M118T30 | 0.01 | -2.05 | 2.40 |  |  |  |  |
| M47T30 | 0.01 | -1.77 | 2.34 |  |  |  |  |
| M134T30 | 0.02 | -2.13 | 2.19 |  |  |  |  |
| M106T30 | 0.01 | -2.16 | 2.11 |  |  |  |  |
| M74T30 | 0.01 | -1.97 | 2.43 |  |  |  |  |
| M89T30 | 0.01 | -1.99 | 2.42 |  |  |  |  |
| M56T30 | 0.01 | -1.78 | 2.37 |  |  |  |  |
| M88T30 | 0.01 | -2.01 | 2.44 |  |  |  |  |
| M116T30 | 0.01 | -2.12 | 2.41 |  |  |  |  |
| M87T30 | 0.01 | -1.94 | 2.39 |  |  |  |  |
| M48T30 | 0.01 | -1.62 | 2.22 |  |  |  |  |
| M76T30 | 0.01 | -1.92 | 2.29 |  |  |  |  |
| M86T30 | 0.01 | -1.98 | 2.43 |  |  |  |  |
| M65T30 | 0.02 | -1.80 | 2.41 |  |  |  |  |
| M64T30 | 0.01 | -1.99 | 2.41 |  |  |  |  |
| M204T30 | 0.03 | -1.22 | 2.08 | 29.85 | N/A |  |  |
| M290T30_1 | 0.02 | -3.21 | 1.66 | 29.92 | N/A |  |  |

CAS, Chemical Abstracts Service; FC, fold change; N/A, not available; *q* value, false discovery rate (FDR)-adjusted *p* value; RSI, reverse search index; RT, retention time; VIP, variable importance in projection; VOC, volatile organic compound.

Table S3: Intra-day precision of hexanal at different concentration levels.

| Concentration level | Replicate 1  (a.u.) | Replicate 2  (a.u.) | Replicate 3  (a.u.) | Mean  (a.u.) | SD  (a.u.) | RSD  (%) |
| --- | --- | --- | --- | --- | --- | --- |
| Low | 5860575 | 5885489 | 5982896 | 5909653 | 64642 | 1.09 |
| Medium | 9666652 | 10204814 | 11831776 | 10602414 | 1130947 | 10.67 |
| High | 20673011 | 22343116 | 23892610 | 22301246 | 1609093 | 7.22 |

a.u., arbitrary units; RSD, relative standard deviation; SD, standard deviation.

Table S4: Inter-day precision of hexanal at different concentration levels.

| Concentration level | Peak area  (Day 1, a.u.) | Peak area  (Day 2, a.u.) | Peak area  (Day 3, a.u.) | Mean  (a.u.) | SD  (a.u.) | RSD  (%) |
| --- | --- | --- | --- | --- | --- | --- |
| Low | 5982896 | 5468469 | 5527325 | 5659563 | 281466 | 4.97 |
| Medium | 10204814 | 8688223 | 7559225 | 8817421 | 1324745 | 15.02 |
| High | 20673011 | 17643133 | 18629085 | 18981743 | 1535557 | 8.09 |

a.u.: arbitrary units; RSD: relative standard deviation; SD: standard deviation.

Table S5: Diagnostic performance of serum CEA, pleural effusion CEA, and hexanal for distinguishing LC-associated MPE from BPE.

|  | Discovery cohort | | | Validation cohort | | |
| --- | --- | --- | --- | --- | --- | --- |
|  | Hexanal | PE CEA | Serum CEA | Hexanal | PE CEA | Serum CEA |
| Sensitivity | 70.30% | 64.80% | 55.70% | 75.00% | 87.50% | 56.30% |
| Specificity | 78.30% | 96.50% | 100.00% | 65.71% | 95.45% | 95.80% |
| Accuracy | 74.40% | 81.10% | 74.30% | 70.15% | 91.30% | 73.20% |

BPE, benign pleural effusion; CEA, carcinoembryonic antigen; LC, lung cancer; MPE, malignant pleural effusion; PE, pleural effusion.

**Supplementary Figures**


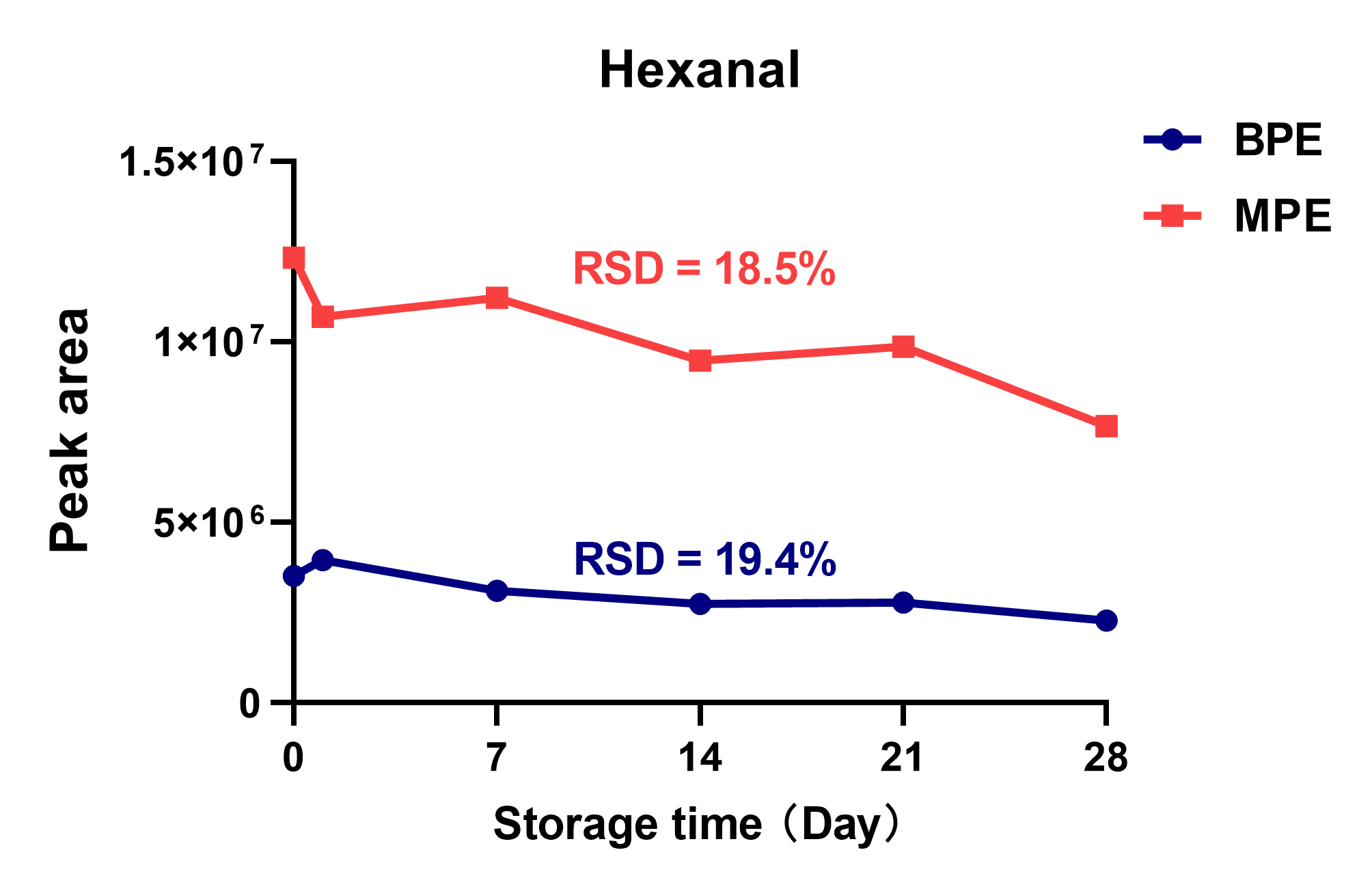


Figure S1: Storage-time stability of hexanal in aliquoted pleural effusion samples stored at −80°C.

BPE, benign pleural effusion; MPE, malignant pleural effusion; RSD, relative standard deviation.


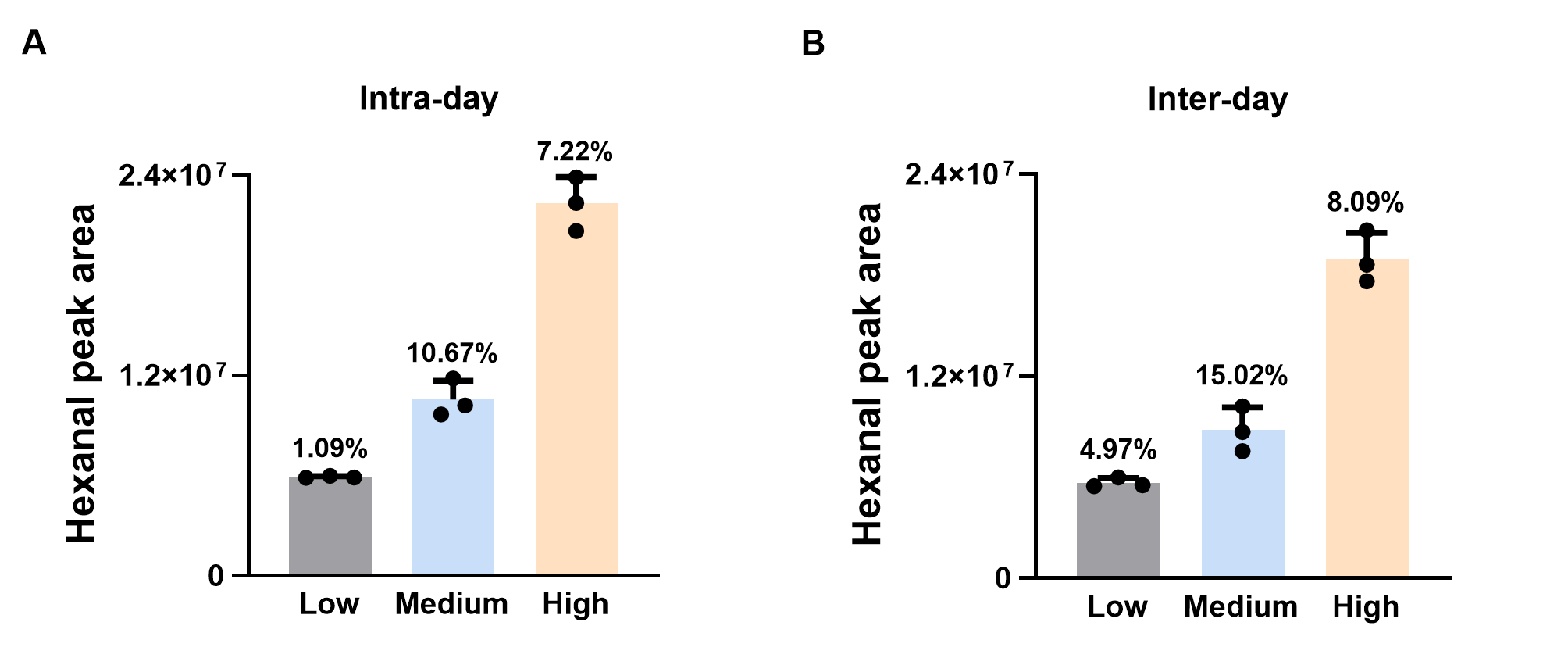


Figure S2: Intra-day and inter-day precision of hexanal measured by HS-SPME-GC–MS.

1. Intra-day precision of hexanal evaluated using three replicate measurements at low, medium, and high concentration levels performed within the same analytical day. (B) Inter-day precision of hexanal assessed across three independent analytical days at corresponding concentration levels using an identical analytical protocol. Bars represent mean ± SD, and each dot denotes one independent measurement (n = 3). Hexanal peak areas are reported in arbitrary units (a.u.).


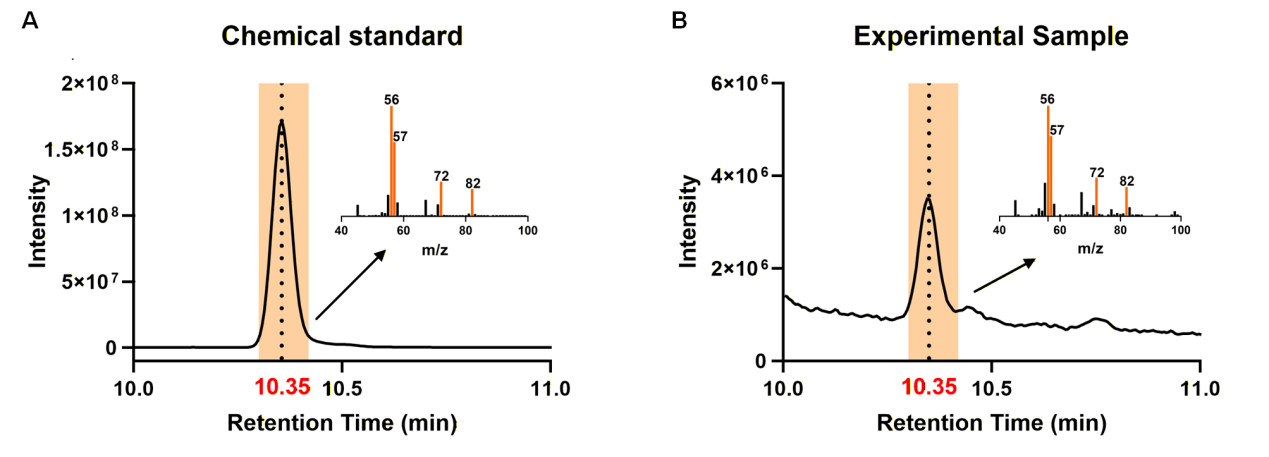


Figure S3: Qualitative validation of hexanal in pleural effusion by GC–MS using an authentic chemical standard.

1. Extracted ion chromatogram (EIC) and representative mass spectrum of the authentic hexanal chemical standard; (B) Corresponding EIC and mass spectrum of hexanal detected in the experimental sample, exhibiting an identical retention time (10.35 min) and highly consistent characteristic fragment ions (m/z 56, 57, 72, and 82).


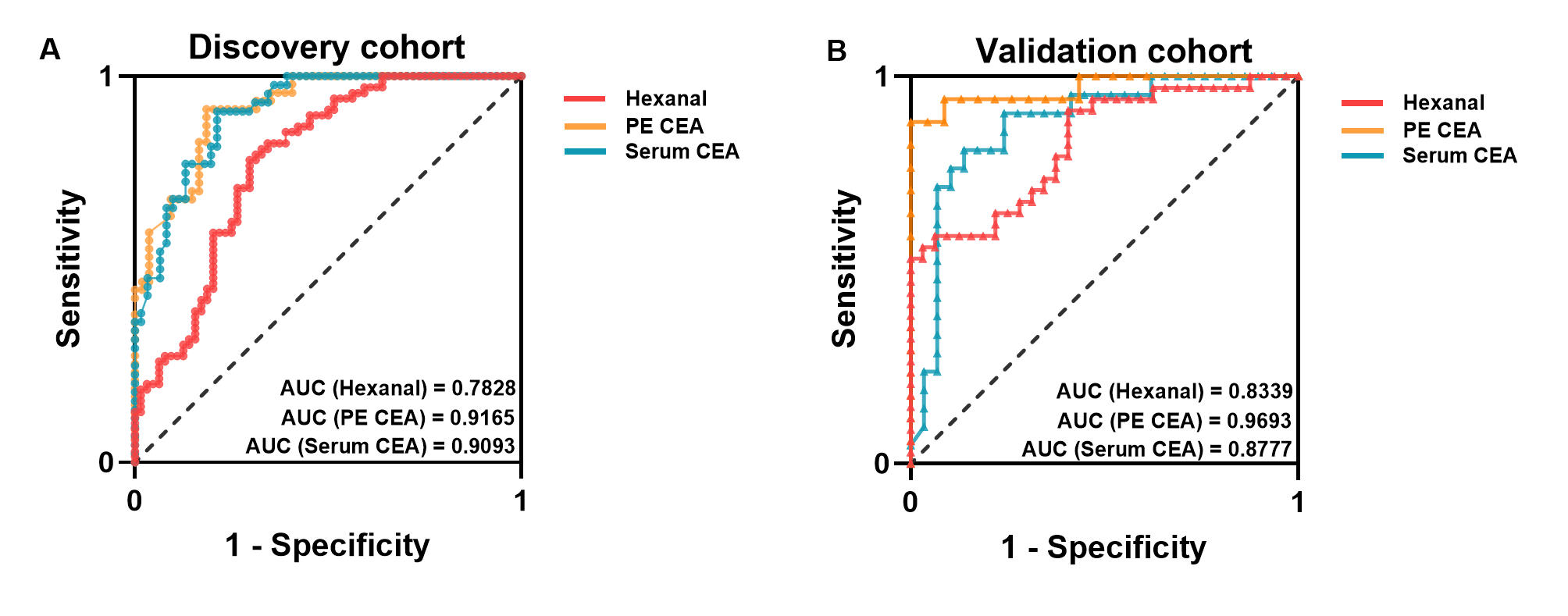


Figure S4: ROC curves of serum CEA, pleural effusion CEA, and hexanal for distinguishing LC-associated MPE from BPE in the discovery and validation cohorts.

BPE, benign pleural effusion; CEA, carcinoembryonic antigen; LC, lung cancer; MPE, malignant pleural effusion; PE, pleural effusion; ROC, receiver operating characteristic.


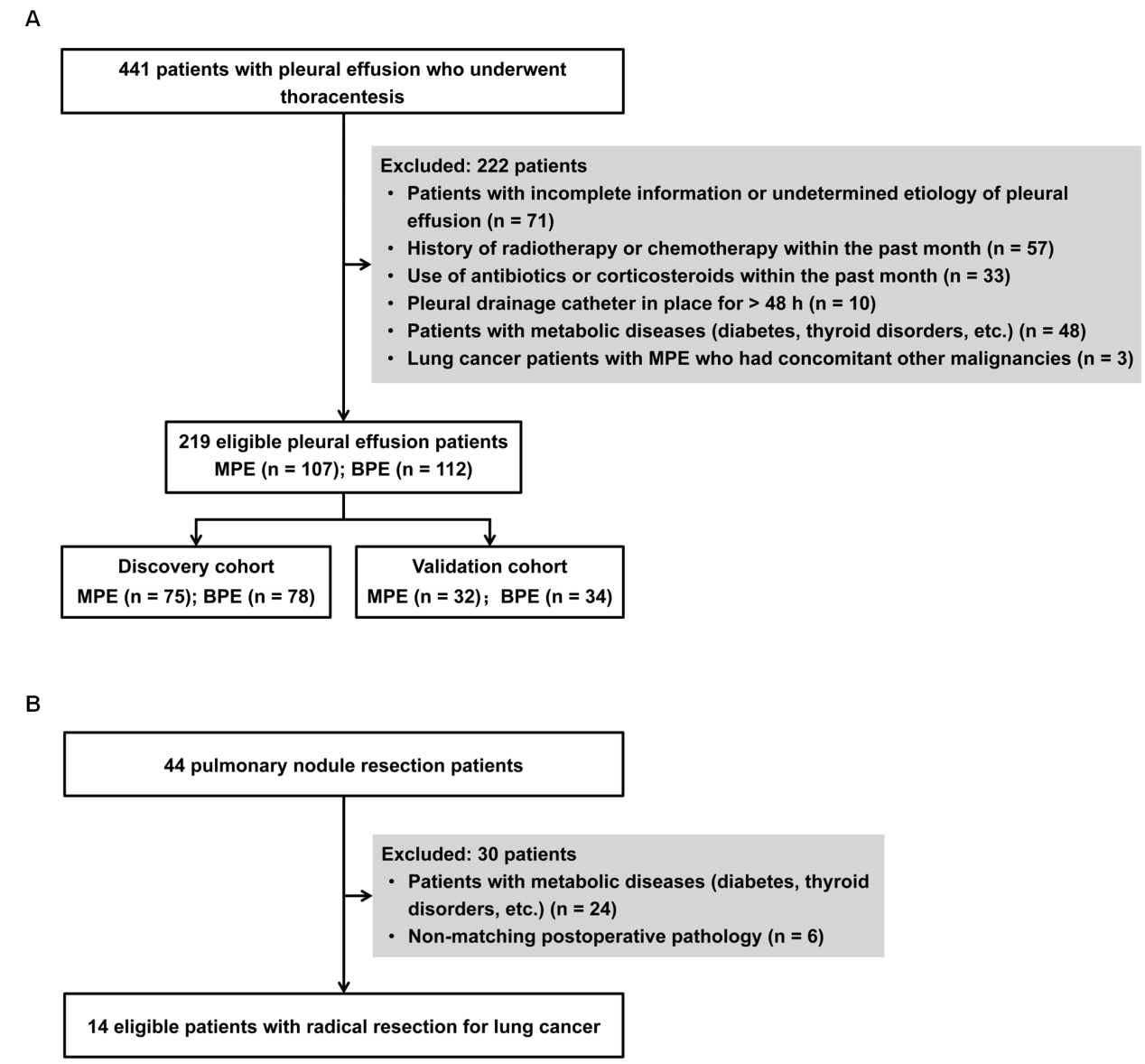


Figure S5: Patient enrollment process for the pleural effusion and tissue cohorts.

1. Enrollment process for patients in the pleural effusion cohort. (B) Enrollment process for tissue cohort patients. BPE, benign pleural effusion; MPE, malignant pleural effusion.


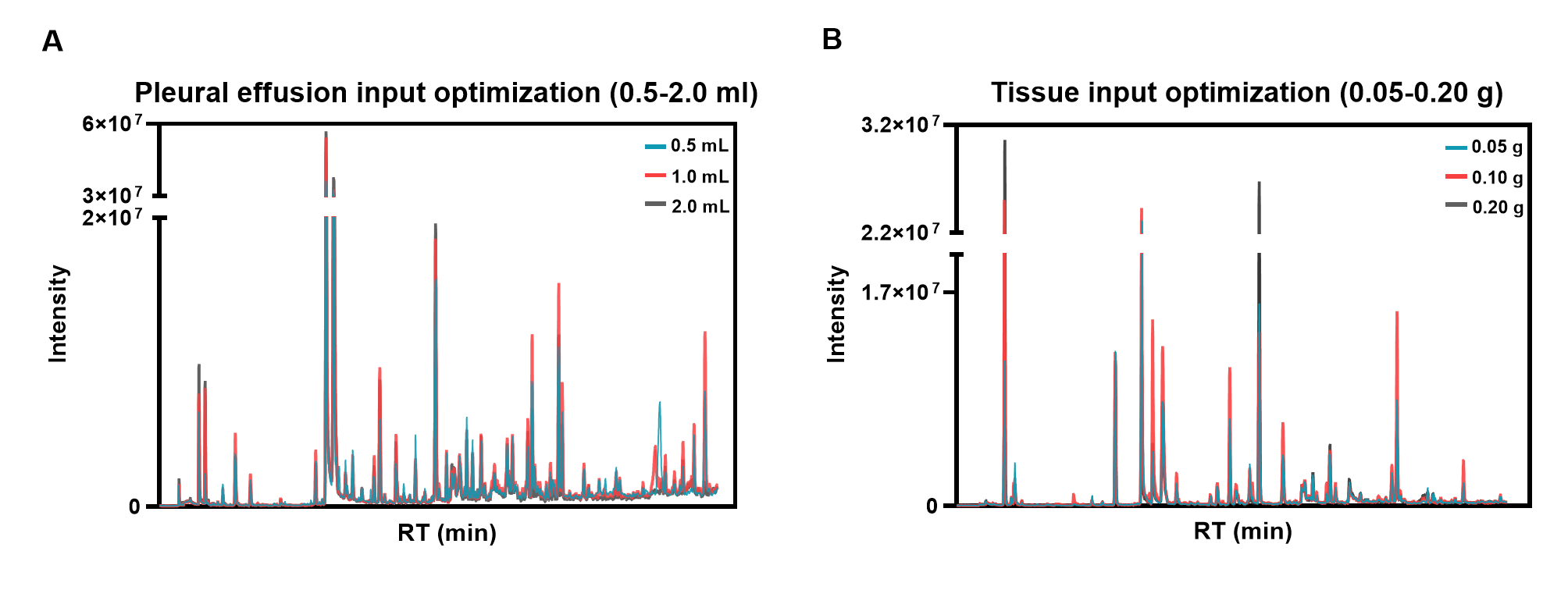


Figure S6: Optimization of sample input amounts for HS-SPME-GC–MS analysis.

1. Representative GC–MS total ion chromatograms of pleural effusion samples at input volumes of 0.5, 1.0, and 2.0 mL; (B) Representative GC–MS total ion chromatograms of lung tissue samples at input amounts of 0.05, 0.10, and 0.20 g. GC–MS, gas chromatography–mass spectrometry; HS-SPME, headspace solid-phase microextraction; RT, retention time; TIC, total ion chromatogram.


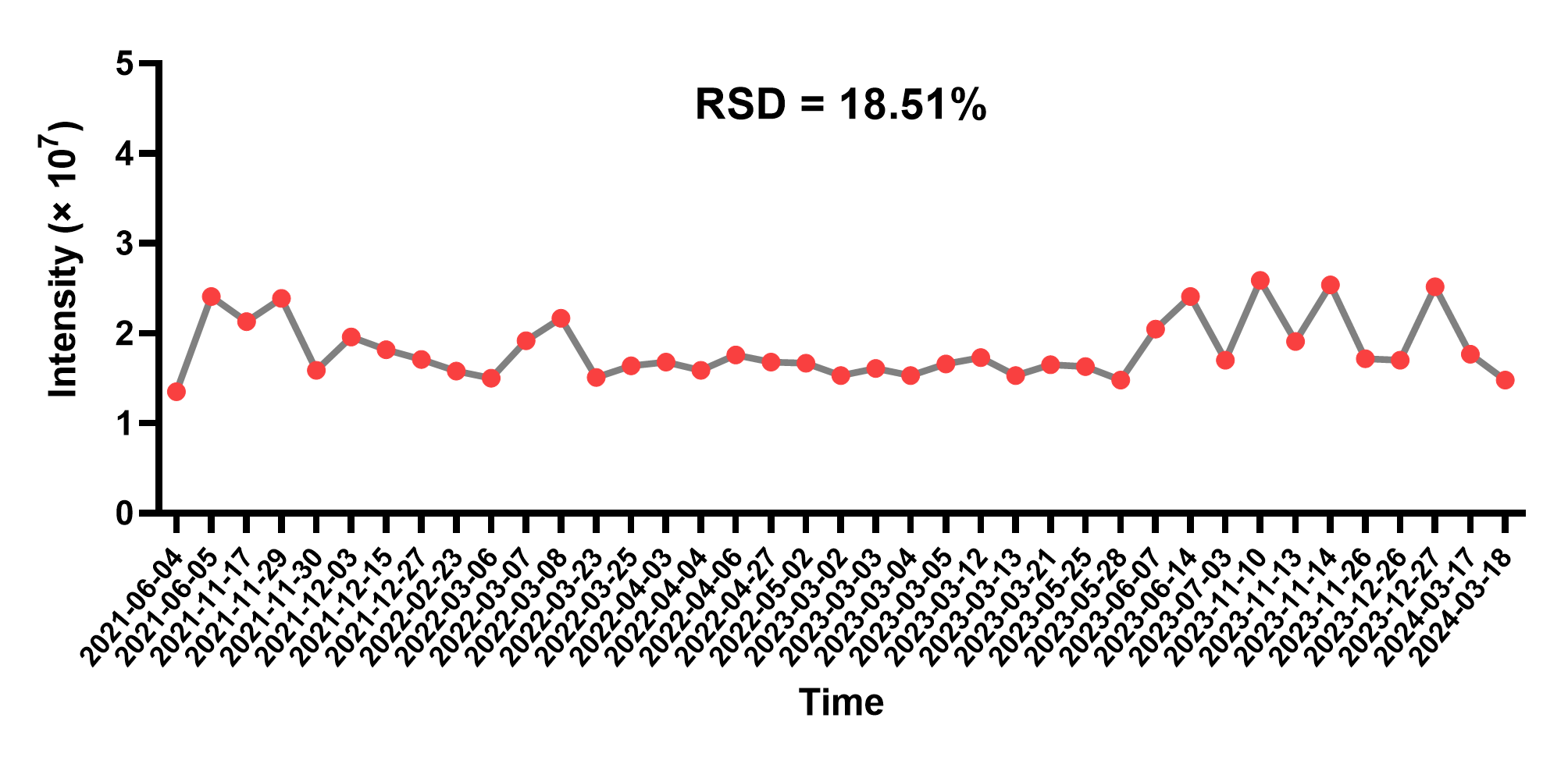


**Figure S7:** Daily PFTBA TIC signal intensity during the GC–MS analytical period.

The TIC signal of the PFTBA standard was recorded once per analytical day to monitor instrument stability. The overall RSD across all analytical days was 18.51%. RSD, relative standard deviation; TIC, total ion chromatogram; PFTBA, perfluorotributylamine.
